# Analog Tensor Processing With Carbon Nanotube In‐Memory Matrix Multiplications for Edge Computer Vision Acceleration

**DOI:** 10.1002/adma.74633

**Published:** 2026-08-13

**Authors:** Jingfang Pei, Lekai Song, Songwei Liu, Yingyi Wen, Yaoqiang Zhou, Yang Liu, Pengyu Liu, Wenyu Cui, Xin Lu, Teng Ma, Zegao Wang, Guohua Hu

**Affiliations:** ^1^ Department of Electronic Engineering The Chinese University of Hong Kong Hong Kong SAR China; ^2^ Department of Applied Physics Hong Kong Polytechnic University Kowloon Hong Kong SAR China; ^3^ School of Physics Beijing Institute of Technology Beijing China; ^4^ Shun Hing Institute of Advanced Engineering The Chinese University of Hong Kong Hong Kong SAR China; ^5^ College of Materials Science and Engineering Sichuan University Chengdu China

**Keywords:** carbon nanotubes, computing‐in‐memory, edge computer vision, tensor core

## Abstract

Computer vision requires intense tensor operations, primarily matrix multiplications, imposing substantial computation demands. Using hardware such as GPU and ASICs for acceleration offers a viable solution. Their application at edge, however, can be constrained by complexity in the computing architecture and incompatibility with analog systems. Here, we prototype an analog tensor core based on carbon nanotube charge‐trapping nonvolatile memory for edge computer vision acceleration. The memory, exhibiting ∼100 linear, symmetric analog weights with fast programming (500 ns) and data processing (1 Mbit/s), enables in‐memory matrix multiplications, facilitating compact tensor core design and operation of vectors and scalars in visual tasks. As demonstrations, the analog tensor core proves three‐dimensional (3D) spatial transformation with an error of <2.81% and edge detection with a signal‐to‐noise ratio of >22 dB, underpinning its potential for accelerating computer vision tasks in, for example, autonomous driving, VR/AR, robot navigation, and industrial automation. Proof‐of‐concept simulation achieves viewfield distortion correction and edge detection of street view fisheye captures by parallel single‐layer tensor processing using our analog tensor core in large scales.

## Graphical abstract

This work prototypes a carbon nanotube‐based analog tensor core that performs in‐memory, parallel visual processing. Integrating non‐volatile memories and compact circuits, the core enables high‐speed analog matrix multiplications and can demonstrate accurate three dimensional (3D) spatial transformation and edge detection. With lightweight design, the analog tensor core is suited for accelerating edge vision tasks in autonomous driving, VR/AR, and industrial automation.

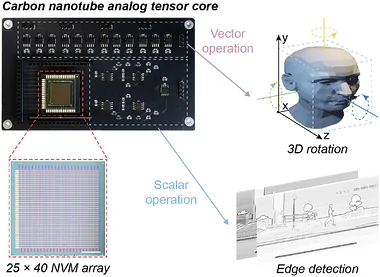

## 1 Introduction

Interpreting and understanding visual information captured by the edge visual nodes is key to computer vision applications, including autonomous driving, VR/AR, robot navigation, and industrial automation [1, 2, 3, 4, 5, 6, 7]. Taking autonomous driving as an example (Figure 1a), an efficient perception of the street view is essential to determine the roadway conditions and recognize the pedestrians and vehicles [8, 9]. To achieve this, the various types of vectors (e.g., spatial location, optical flow) and scalar (e.g., pixel intensity, frame rate) data comprised need to be processed in time [10, 11]. Current approaches use feedforward neural networks, for example, convolutional neural networks, to perform the vector and scalar computation tasks [12, 13]. However, accomplishing these tasks involves transport of large volumes of visual data from the edge to the remote cloud computing units and the subsequent data processing via the multilayer matrix multiplications [14]. This, as a result, limits the computing efficiency and incurs latency [15], while minuscule delays in perception in autonomous driving can risk the driving safety [16]. Driven for faster, more efficient computer vision, solutions are explored to accelerate the computation, particularly toward edge deployment [15, 17].

**FIGURE 1:**
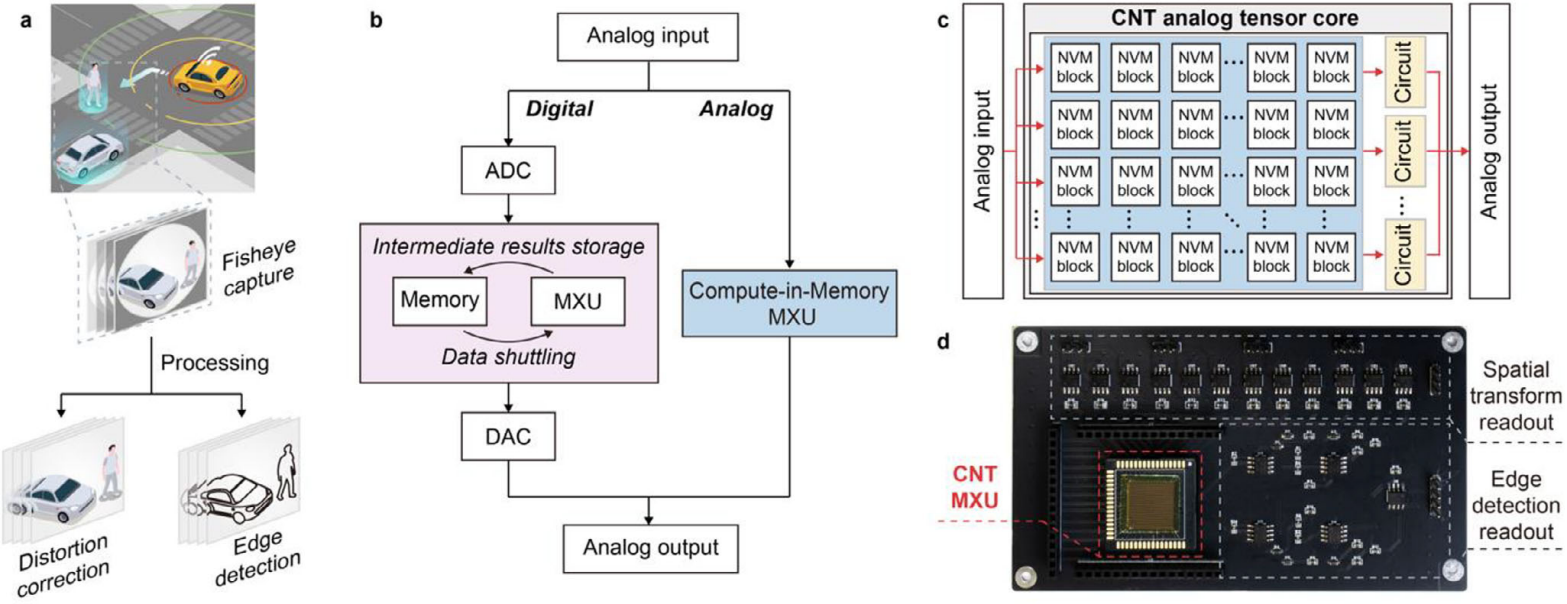
Accelerating computer vision in autonomous driving. (a) Computer vision tasks in autonomous driving, showing viewfield distortion correction and edge detection of fisheye captures. (b) Architectures of digital and analog accelerators, where the analog approach avoids digital‐analog conversion and memory‐MXU data transfer. (c) Architecture of our CNT analog tensor core, packaged with an analog MXU integrating CNT NVMs and the peripheral vector and scalar processing modules. The MXU is programmed in blocks to perform parallel vector and scalar matrix multiplications in tensor processing. (d) Prototype of an analog tensor core, packaged with a CNT MXU and the readouts for spatial transformation and edge detection operations. The MXU integrates 25 × 40 CNT NVMs in crossbar structure.

Currently, hardware‐based solutions using general proposed graphics processing units (GPUs) and application specific integrated circuits (ASICs) emerge promising for computer vision acceleration [18, 19]. Tensor processing units (TPUs), as ASICs, are of particular interest. A TPU generally consists of a matrix multiply unit (MXU) and peripheral vector and scalar processing modules. During operation, incoming vector and scalar data streams are distributed into small blocks, enabling highly efficient parallel matrix multiplication [20, 21]. For example, Google's TPU *Trillium* showcases a peak performance of 1836 tera operations per second through parallel computing using hundreds of thousands of tensor processing units [20]. Compared to GPUs with equivalent computing power, TPUs can reduce the energy consumption by approximately 5–10 times [20, 22]. However, pushing these accelerators toward the edge remains challenging due to the intense computation workload and the highly constrained hardware resource at edge. The bottleneck is that, limited by their computation architecture (Figure 1b, left), the computation is separated from the data storage and as a result, the data has to be transferred constantly to the MXU to accomplish the computation [14]. This limits the bandwidth and incurs latency. In addition, conforming to digital computing, the data has to be converted using analog‐to‐digital and digital‐to‐analog (AD/DA) converters, leading to excessive computation and hardware cost [23]. With the rise of nonvolatile memory (NVM) and computing‐in‐memory technology, developing MXU using NVMs and implementing the computation conforming to computing‐in‐memory show promise to address the challenges [11, 23]. In this approach (Figure 1b, right), the NVM MXU with compact vector and scalar processing modules can be potentially deployed at the visual edge hardware, and the analog visual data as captured can perform local computing without AD/DA conversions or data transfer. Despite significant progress in computing‐in‐memory architecture and system engineering, end‐to‐end hardware implementations built on emerging nonvolatile memory platforms remain challenging, particularly when scalable fabrication, circuit integration, and task‐level implementation are considered together.

In this work, toward computer vision acceleration at edge, we develop an analog tensor core using carbon nanotube (CNT) NVM transistors. The NVMs, based on charge‐trapping, present linear and symmetric programmable memory weights (with ∼100 weights programmable at a speed of ∼500 ns) and a high‐speed data processing (over ∼1 Mbit/s). These characteristics enable the design of a compact analog tensor core for tensor processing (Figure 1c). In this architecture, an NVM crossbar‐based MXU is integrated with peripheral vector and scalar processing modules. By programming the array in blocks, parallel in‐memory multiplication of vector and scalar inputs can be performed. For demonstration propose, we fabricate a PCB‐level task‐oriented computing system as a small‐scale analog tensor core prototype (Figure 1d, Figures S1, and S2) and assign the prototype to perform analog tensor processing of visual data. We prove vector spatial transformation (with error <2.81%) and scalar edge detection (with signal‐to‐noise ratio >22 dB). The tensor core with this tensor processing capability, along with its lightweight and custom vector and scalar processing circuit designs, holds the potential to accelerate real‐world computer vision at edge. We explore large‐scale tensor processing of street view fisheye captures in autonomous driving using our analog tensor core via simulation, and prove successful single‐layer tensor processing of viewfield distortion correction and edge detection.

## 2 Results

### 2.1 Analog Matrix‐Multiply Unit

In design of an analog tensor core (Figure 1c), MXU as the processing core expects to perform vector and scalar matrix multiplications with peripheral vector and scalar processing modules. This in turn requires multi‐level, programmable memory weights in the NVMs, so as to allow the multiplication function programming [11]. Here we develop NVMs using CNTs based charge‐trapping transistors following our previous report [24]. Briefly, semiconducting single‐walled CNTs sorted by polymer poly[n‐(1‐octylnonyl)‐9h‐carbazole‐2,7‐diyl] (PCz) (Figure S3) are deposited on silicon wafer by dip coating (Figure S4) and used to fabricate bottom‐gate bottom‐contact transistors using standard photolithography (Figure S5). Upon charge trapping, CNTs with high carrier mobility and concentration as well as large specific surface area can realize non‐volatile memory with precisely programmable weights [24].

Figure 2a shows the NVMs on a silicon wafer and, specifically, the analog MXU designed in our work, where an array of 25 × 40 CNT NVMs is integrated in crossbar structure (Figure 2b,c). To validate that the NVM array can work as MXU and perform the multiplication operations, we conduct a block test of 20 × 20 NVMs. The results show a non‐volatile memory with a memory window of ∼10.4 V (Figure 2d,e and Note S1) and, notably, statistical analyses prove a 100% success rate in the fabrication with high device‐to‐device uniformity (Figure 2f,g). See also Figure S6 for the memory non‐volatility characteristic. Further memory programming by single gate pulse (duration 1 ms) of varying amplitudes demonstrates ∼100 stable, easily distinguishable memory weights, and these weights establish a linear relation with the gate bias (*R* ^2^ of 0.981 in the linear programming region; Figure 2h). The NVMs with the linear programmable, multi‐level nonvolatile memory weights, as well as the high success rate and uniformity in wafer‐scale fabrication, prove the capability to develop MXU. Importantly, the linear, wide‐range memory weights are key for analog data processing, in contrast to digital data processing, in performing analog tensor processing. Conforming to tensor processing, the MXU can be programmed in blocks to distribute, for example, 104 3 × 3 matrix‐multiply and 40 5 × 5 convolutional kernels. Note that to allow precise programming of the individual NVMs among the interlayer crossbar, an insulating barrier layer of photoresist SU‐8 is introduced (Figure 2c). SU‐8 is widely used in microfabrication as an insulating, passivation, and packaging material owing to its excellent electrical insulation, chemical stability, and process compatibility [25, 26, 27, 28]. As the barrier layer, SU‐8 exhibits a long‐term stability and shows no obvious degradation during CNT NVM fabrication and the subsequent electrical testing in our work.

**FIGURE 2:**
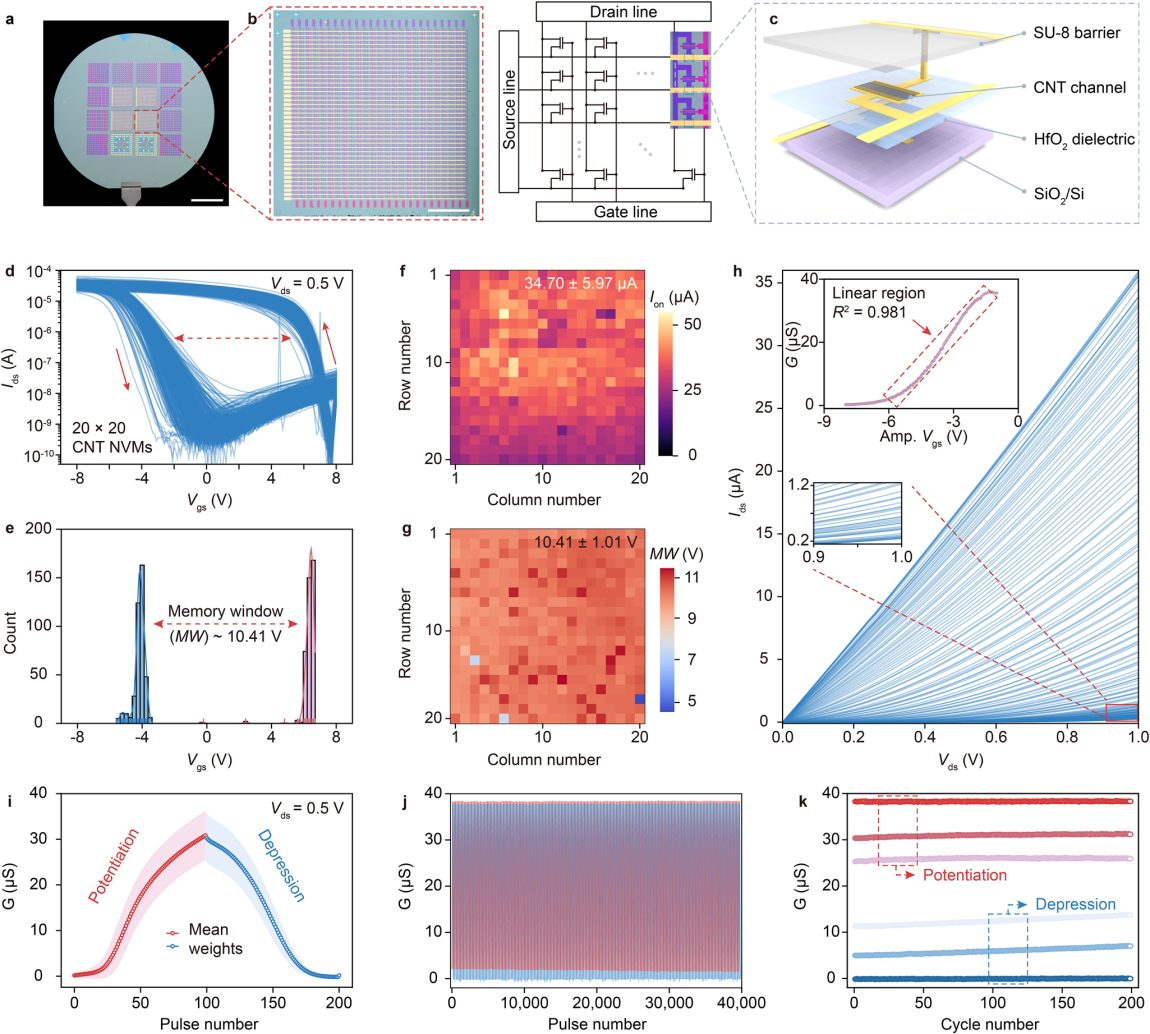
Analog matrix‐multiply unit (MXU). (a) CNT NVM transistors fabricated on a 4‐inch silicon wafer. (b) An analog MXU integrating 25 × 40 CNT NVMs in crossbar structure, and the MXU circuit diagram. Scale bars: (a) 2 cm, (b) 2 mm. (c) Structure of a crossbar node. (d) Transfer characteristics tested from a 20 × 20 NVM block. (e) Statistical normal distribution of the threshold voltages (*V*_th_). The positive and negative *V*_th_ are 6.23 ± 0.99 V and −4.15 ± 0.55 V, respectively. Heatmaps of the measured (f) on‐state current and (g) memory window. The on‐state current and memory window (*MW*) are 34.70 ± 5.97 µA and 10.41 ± 1.01 V, respectively, proving a high device‐to‐device uniformity. (h) Memory weight programming of a typical NVM by a set of long single *V*_gs_ pulses of varying amplitudes (from −1 V to −8 V, step 0.05 V, duration 1 ms). The NVM is reset by a *V*_gs_ pulse (8 V, 5 ms) before each programming. The inset shows linear weight programming across ∼100 states, with the coefficient of determination *R* ^2^ >0.98. (i) Potential‐depress weight programming of 36 randomly selected NVMs using ultrafast pulse trains (duration 500 ns, interval 500 µs) of varying pulse amplitudes, showing a linear and symmetric programming behavior consistent in these sampled devices. Each programming cycle is performed by 100 positive *V*_gs_ pulses followed by 100 negative *V*_gs_ pulses. The dots and background represented the mean weights and standard deviations, respectively. See the ultrafast pulse train waveform in Figure S8. (j) Pulsed potential‐depress weight programming of a typical NVM through 200 cycles, and (k) the cycle‐to‐cycle evolutions of 3 randomly selected weights in potentiation (red) and depression (blue). The cycle‐to‐cycle weight programming variation is <0.16%, proving a stable memory weight programming.

Given the linear analog memory weight programming capability of the NVMs, and that a high‐speed programming is instructive to MXU operations in tensor processing, we propose to use fast potentiation and depression pulse trains to adaptively program the NVMs. However, note that fast pulse trains of constant amplitudes (amplitude 3 V, duration 10 µs, interval 990 µs) give a poor linearity (Figure S7). We address this by using ultrafast pulse trains (duration 500 ns, interval 500 µs) of varying pulse amplitudes, where the pulse amplitude along with the pulse number are adaptively configured to set the weight in a linear manner (Figure S8). The exhibited nonlinearity factor is down to ∼1.17 and −1.44 for potentiation and depression, respectively (Figure 2i). In addition, note that with a large pulse duration, the device can achieve a higher switching ratio, that is, a wider weight programming window (Figure S9). As such, arising from CNT‐cross‐connection charge trapping by the sorting polymer PCz [24], a linear memory weight programming of the NVMs with ultrafast pulse trains is realized. In addition, this pulsed programming strategy ensures stable memory weights, for example, cycling pulsed potentiation–depression weight programming achieves highly stable multi‐state weights, with a cycle‐to‐cycle variation of ∼2% (Figure 2j,k). To specify, Figure 2j assesses and demonstrates the reproducibility of analog weight reprogramming under repeated potentiation/depression pulse trains, while Figure 2h defines the accessible conductance range under single‐pulse programming. This repeatability is further proved in Figure 2k through the cycle‐to‐cycle evolution of selected intermediate conductance states. Besides weight programming, a fast and stable data processing is instructive to MXU operations. This is ensured as well by the NVMs, where the NVMs demonstrate a pulsed drain‐source signal processing with a speed of 1 MHz (Figure S10) and over a volume of 10^7^ pulses (Figure S11), meaning that the MXU can allow for a data processing at 1 Mbit/s per channel and over a volume of 10^7^ bits in one single operation.

Quantitative analysis of the analog programming characteristics using a ±1σ‐based state‐separability criterion shows that the CNT NVM can achieve up to 72 effective conductance states, corresponding to 6.17 effective bits, during potentiation; and 61 effective states, corresponding to 5.93 effective bits, during depression (Figure S12, Table S1, and Note S2). Using pulsed programming strategy, here we estimate the energy consumption of our NVMs is down to ∼3.98 fJ for one weight programming and ∼30.8 pJ for one bit data processing (Note S3 and Table S2). This energy consumption meets the requirements for low‐power edge data processing and applications, making our NVMs promising for realizing computing‐in‐memory architectures, including tensor processing. However, we must note that in practical integrated circuits and tensor processing chips, the energy consumption beyond the NVMs can be greatly impacted by the specific peripheral circuit designs and semiconductor manufacturing process technologies. Our study primarily focuses on the energy consumption for operating the NVMs instead of the peripheral circuits in tensor processing.

### 2.2 Experimental Vector Operation for Spatial Transformation

In practical computer vision, the visual information as captured can be projected as pixels in a distorted space, with their spatial positions described with a displaced 3D coordination matrix (*x_n_*, *y_n_*, and *z_n_*). This distorted coordination matrix, essentially determining the geometric structure, shape, and layout of the objects, often needs a correction for the successful visual information understanding [29, 30]. The correction can be done by spatial transforming the coordinates [31]. This type of vector operation, though fundamental, poses challenges to the current computing approaches, as a pixel‐wise matrix multiplication of large volumes of coordinates can result in redundant computation workload and latency [32].

As we discuss, tensor processing, particularly in analog, is an effective acceleration approach for matrix multiplication. Here we study the feasibility of using our analog tensor core (Figure 1d) to accelerate spatial transformation of 3D coordination matrix. To conveniently illustrate the idea, we take the cube in Figure 3a as an example. The cube can be represented by eight vertex coordinates (*x_n_*, *y_n_*, *z_n_*) that can be then multiplied by a 3 × 3 transformation matrix to yield a new set of vertex coordinates ($x_{n}^{\prime },\;y_{n}^{\prime },\;z_{n}^{\prime }$). ($x_{n}^{\prime },\;y_{n}^{\prime },\;z_{n}^{\prime }$) can be used to render the transformed cube. The transformation matrix, termed *spatial operator*, consists of spatial angular correction information and is programmed to perform the target transformation operations. Conforming to the spatial transformation operation, our analog tensor core (Figure 1d) is designed to connect the MXU with back‐end transimpedance amplifier (TIA) readout (Figure 3a). When in operation, 3 × 3 NVMs are programmed in blocks with the target angular information to distribute the *spatial operation* units. The input (*x_n_*, *y_n_*, *z_n_*), as captured in analog, is mapped directly as the parallel inputs *V*_*in*1_, *V*_*in*2_, and *V*_*in*3_ to the MXU, while the summation read from the TIAs is subsequently remapped as analog output ($x_{n}^{\prime },\;y_{n}^{\prime },\;z_{n}^{\prime }$) to render the spatial transformation.

**FIGURE 3:**
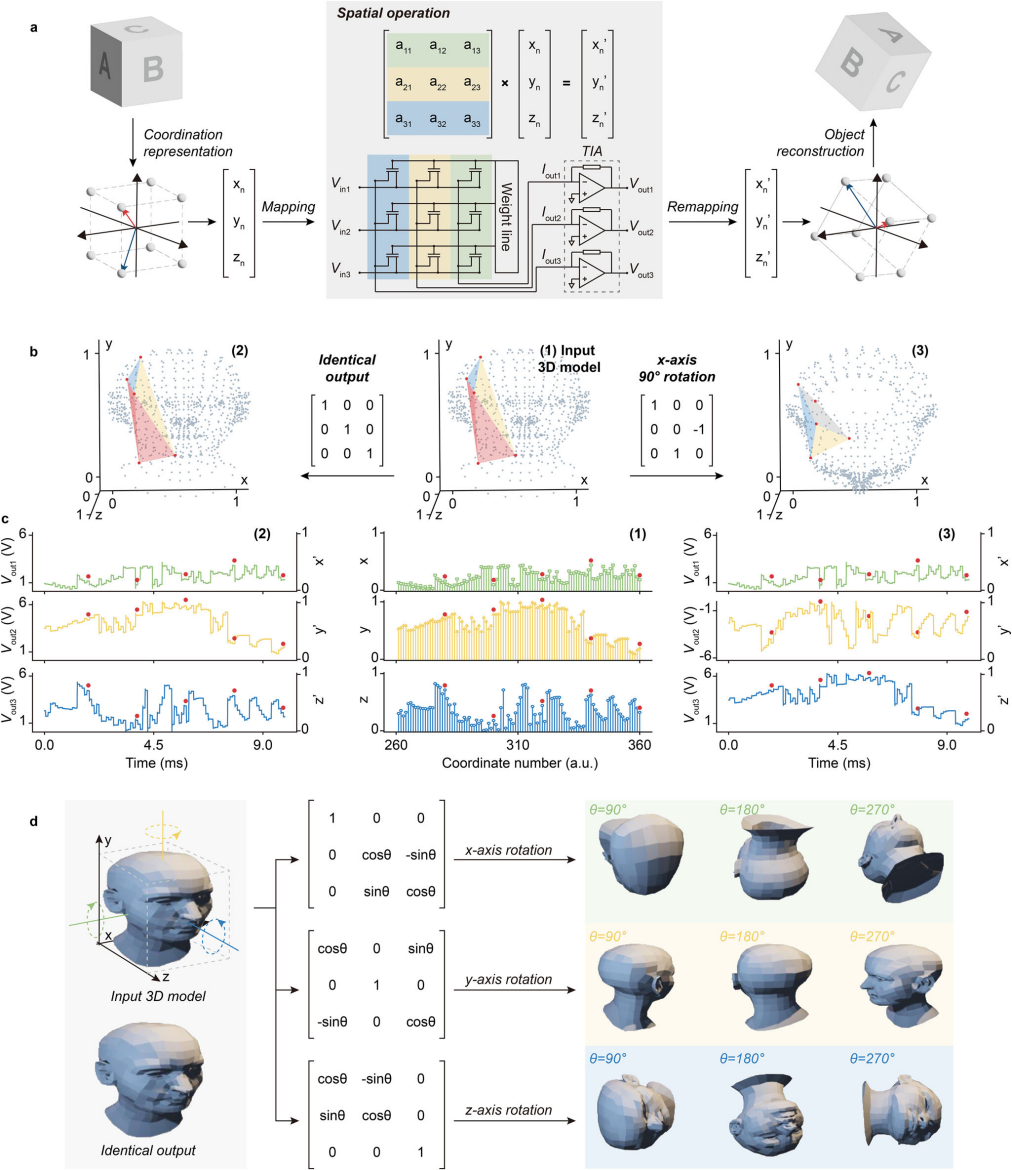
Experimental vector operation for spatial transformation. (a) Principle of spatial transformation, where the vertex coordinates of the cube are extracted and computed with the spatial operator via matrix multiplications, followed by coordination reconstruction to render the transformed cube. The spatial operation is implemented using 3 × 3 programmed NVMs in conjugated with TIA readout. (b) Cloud of 867 vertex coordinates of a sculpted human head model – 1) the input, 2) after an identical transformation, that is, 0° rotation around *x*‐, *y*‐, and *z‐*axis, and 3) after 90° rotation around x‐axis. (c) The *x*‐ *y*‐, and *z*‐ coordination of the selected 100 coordinates – 1) the input, 2) after an identical transformation, and 3) after 90° rotation around *x*‐axis. The voltage signals, *V*_out_ read from the TIA readout to render the transformed model, are shown in parallel. See also Figure S13 for the coordination of the 867 coordinates. In (b) and (c), 5 coordinates are randomly selected to visualize the successful transformation operations. (d) Omnidirectional rotation of the model. The model rotates 90°, 180°, and 270° around the *x*‐, *y*‐, and *z*‐axes, and the transformed model is reconstructed using the voltage signals *V*_out_ read from the TIAs. See Figure S14 for omnidirectional rotation of the vertex coordinates of the model. The spatial operators are all configured using 3 × 3 programmed NVMs. See Movie S1 for the omnidirectional rotation of the model.

We experimentally study the efficacy of our analog tensor core in performing vector operation for spatial transformation using a sculpted human head model. This model, common in 3D modelling, consists of 867 vertex coordinates (Figure 3b, (1)). Following the above operation principle, these vertex coordinates are encoded as parallel x‐, y‐ and z‐ inputs (Figure 3c, (1)) to the MXU, while the summed multiplication outputs read from the TIA readout render the transformed model. As such, programming the MXU as an identity spatial operator and a spatial operator for 90° rotation around x‐axis allows for an identical transformation (i.e., 0° rotation around *x*‐, *y*‐, and *z*‐axis, Figure 3bc, (2)) and a 90° rotation transformation around *x*‐axis (Figure 3b,c, (3)), respectively. See also Figure S13 for the full inputs and outputs in these two transformations. We select five random coordinates to form a quadrangular cone in space, and a brief observation of the transformed quadrangular cones indicates successful transformations (Figure 3b). To have quantitative evaluation, taking one of the coordinates (0.164, 0.696, and 0.713) as the example, we estimate the percentage and relative *Euclidean* errors [33] associated with both the transformed identical coordinate (0.160, 0.700, and 0.715) and 90° rotated coordinate around *x*‐axis (0.161, 0.287, and 0.693). Errors of <1% are proved, demonstrating that our analog tensor core can successfully perform spatial transformation. Note that the overall error is <1.12% for the quadrangular cone, and <2.81% for the sculpted human head model (Tables S3 and S4).

Beyond the above two particular spatial transformations, we show in Figure 3d (see also Figure S14) that the MXU can be freely programmed to render other transformations, for example, 90°, 180°, and 270° omnidirectional rotations of the model around the *x*‐, *y*‐, and *z*‐axis. See Movie S1 for the visualized transformations. It should be noted that the transformation matrices used in this 3D rotation demo are selected for proof‐of‐concept functional verification and involve only coefficients of +1, −1, and 0. This does not represent a fundamental limitation of the CNT NVMs, of which multilevel conductance programmability has been demonstrated in Figure 2. For dynamic 3D rotation tasks, the coefficients (i.e., the weight of the CNT NVMs) may be programmed freely (beyond +1, ‐1, and 0) and dynamically to suit the specific 3D rotation tasks. Given the fast programming (down to 500 ns; Figure S8) and data processing (1 Mbit/s; Figure S10) of the NVMs, each of the transformations can be easily done in 0.1 ms, that is, in principle, a transformation speed of 10 000 fps in large‐scale tensor processing. This estimated throughput of 10 000 fps corresponds to an idealized situation, assuming a full parallel execution of all required operations for a frame by a large‐scale kernel, that is, 0.1 ms per frame. In a practical imaging system, the achievable frame rate will additionally depend on sensor readout, buffering, control logic, and peripheral latency. Nevertheless, the investigation fully verifies the capability of our analog tensor core in performing vector spatial transformation and that, particularly, our analog tensor core can be freely programmed to accommodate target transformation operations. This lays the foundation of using our analog tensor core in performing viewfield distortion correction in practical computer vision.

### 2.3 Experimental Scalar Operation for Edge Detection

The visual information comprises distinctive scalar feature cues, for example, pixel intensity, colors, and frame rate, of the objects alongside their projected spatial coordinates. A successful understanding of the visual information and perception of the objects in practical computer vision demands an efficient processing of these scalar feature cues [29]. Note that the terms “vector” and “scalar” refer to the form of the visual information being processed, rather than the different underlying hardware primitives. Specifically, the spatial coordinate information inherent in the visual data is treated as vector‐type information, whereas the pixel intensity is treated as scalar‐type information. Again, as discussed, the conventional approaches using feedforward deep neural networks to perform the task can pose computation challenges and latency [32].

Having proved vector operation for spatial transformation, we now study the capability of our analog tensor core to implement parallel scalar tensor processing for edge detection. Edge detection is an image processing technique widely employed to extract the feature cues [34]. It can be typically achieved by calculating the intensity difference between the neighboring pixels using convolution [24]. For example, two convolution kernels, termed *Roberts* operators, can be used to traverse the pixels in the viewfield via dot product to yield horizontal (*g*_x_) and vertical (*g*_y_) gradients that can be then normalized and summed to render the edges, that is, *edge*  = |*g*_x_|  + |*g*_y_| (Figure 4a). Following the principle, to implement the *Roberts* operator for edge detection, our analog tensor core (Figure 1d) is designed to connect the MXU with two parallel compact back‐end circuits (Figure 4a). When in operation, two parallel 2 × 2 NVMs (i.e., two 4 × 1 CNT NVM array) are programmed in blocks to distribute the *Roberts* operation units, where the NVMs are readily programmed by the *Roberts* operator weights, and will be sequentially used to process different pixel windows of the image/video stream. The intensity of the pixels, encoded as analog scalars in grayscale, is input to the MXU in parallel to perform multiply‐accumulation. The summation read from the MXU via the back‐end circuits renders the edges (see implementation in Note S4).

**FIGURE 4:**
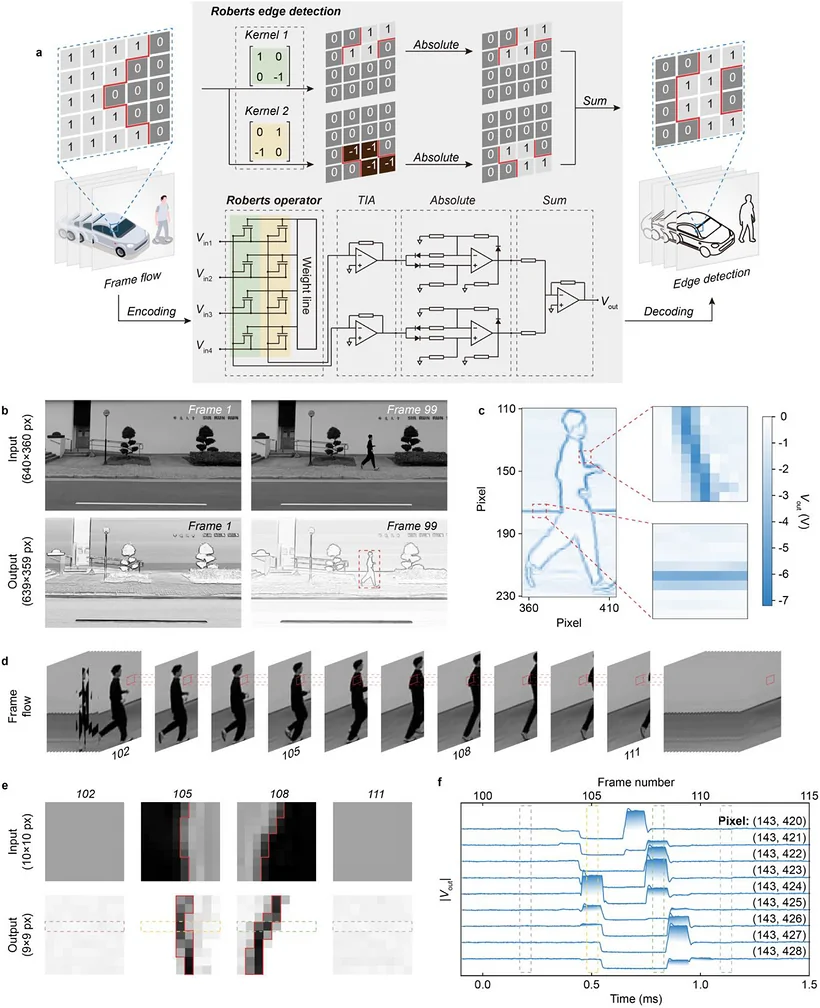
Experimental scalar operation for edge detection. (a) Principle of *Roberts* edge detection, where the pixel intensities are extracted and processed using *Roberts* edge operator via matrix convolution, followed by decoding to output the edges. The *Roberts* edge operator is implemented with two 4 × 1 programmed NVMs in conjugated with back‐end circuits. (b) Input frames in grayscale, and the output frames with the edges detected. The two frames, that is, frame 1 and frame 99, are from a video stream captured from a real scenario. (c) Voltage signals *V*_out_ read from the back‐end circuits to render the edges detected for the pedestrian in frame 99. Both the horizontal and vertical edges of the pedestrian are detected. (d) Real‐time edge detection of the video stream from frame 86 to frame 135, with a region of 10 × 10 pixels selected to study the edge evolution of the moving pedestrian. (e) 4 frames are selected to show the detailed edge evolution before and after the real‐time edge detection operation. See also Figure S17 for the full edge evolution across the 50 frames. (f) Voltage signal *V*_out_ flow read from the back‐end circuits for the selected pixels, with a signal peak‐to‐background ratio of 14.44, proving successful real‐time edge detection. See Movie S2 for the real‐time edge detection.

We study the efficacy of our analog tensor core in detecting the edges of a moving pedestrian in a video stream captured from a real scenario. The video stream consists of 168 frames in 30 fps and 640 × 360 pixels. Here we take two typical frames, frame 1 and 99, to examine the detection capability (Figure 4b). As shown, all the objects in the frames, including the pedestrian and the surrounding building and plants, are separated with their edges clearly rendered after the operation. After edge detection, the standard deviation of the pixel intensities significantly decreases from 45.85 to 38.09 for frame 1, and from 46.46 to 27.28 for frame 99 (Figure S15), proving the effectiveness of the edge detection using our tensor core. To further quantify the detection performance, we present in Figure 4c the edge details of the pedestrian in frame 99, where clear vertical and horizonal edges are illustrated. The signal‐to‐noise ratio (SNR) and dynamic range for the vertical and horizonal edges are estimated as 22.69 and 22.64 dB, and 30.13 and 27.85 dB, respectively (Figure S16). This approaches the industrial standard for real‐world applications. In addition, the mean contrast in the pixel intensity at the edges are substantially improved after the detection operation (Figure S17), further proving the efficacy of our analog tensor core in performing edge detection.

Though successful, the frames in practical computer vision scenarios are captured in timeline, that is, edge detection demands real‐time frame‐to‐frame processing. To demonstrate the real‐time processing capability of our analog tensor core, we present in Movie S2 a 50‐frame video stream showing detection of edges of the moving pedestrian. The video stream is encoded into 10,000 fps to demonstrate high‐speed visual data processing using our analog tensor core. Figure 4d highlights the detection region, while Figure 4e presents the reconstructed edges in the four selected frames. As observed, the edges as detected in these four frames clearly describe the states where the pedestrian enters and leaves the viewfield. See Figure S18 for the detection details in the full 50 frames. Similarly, we estimate the SNR and dynamic ratio (Figure S19) as well as the mean contrast (Figure S20) of two typical frames, that is, frame 105 and 108 (Figure 4e), and prove the efficacy of our analog tensor core in performing real‐time edge detection. To study the real‐time processing capability further, we exam the voltage signal *V*_out_ flow that renders the edges in the selected pixels (Figure 4f and Figure S21). Here the signal peaks render the edges, while the amplitudes determine the intensity. A signal peak‐to‐background ratio of 14.44 through the timeline well presents the real‐time processing capability of our analog tensor core. The investigation proves the feasibility of using our analog tensor core to implement parallel scalar tensor processing for edge detection. The energy consumption of the scalar operation hardware is estimated as ∼1.08 µJ per operation (Note S5, Table S5, and Figure S22), proving a low‐power data processing. Again, given the fast data processing speed of the NVMs, the edge detection operation is implemented at a signal frequency of 10 kHz. As we discuss above, this can give an idealized upper‐bound video stream processing speed of 10 000 fps for a fully parallelized large‐scale tensor processing, fulfilling the requirements of common computer vision tasks [35].

### 2.4 Large‐Scale Tensor Processing

As discussed, tensor processing shows promise to perform parallel matrix multiplications of the various types of vector and scalar quantities comprised in the visual data [12, 20, 21]. Taking autonomous driving as an example again (Figure 5a), the visual data as captured by fisheye lens needs to be processed in time to perform tasks from viewfield distortion correction to feature cure extraction and the subsequent object recognition and decision‐making, in order to ensure the driving safety [29, 30]. Conforming to tensor processing, the vector (e.g., spatial location) and scalar (e.g., pixel intensity) quantities shall be extracted and distributed to perform the parallel matrix multiplication tasks (i.e., the correspondingly viewfield distortion correction and edge detection).

**FIGURE 5:**
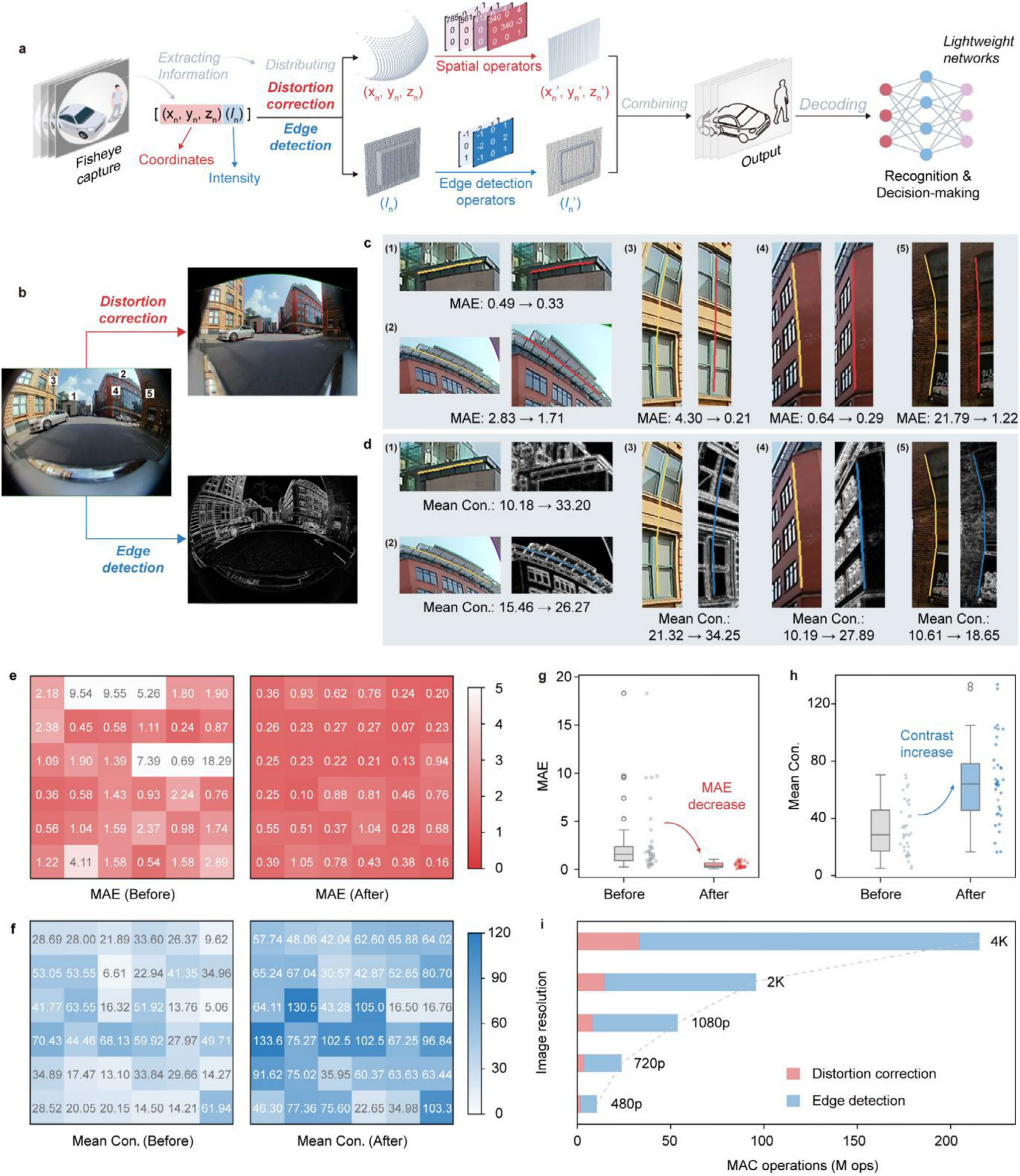
Large‐scale tensor processing accelerating computer vision tasks in autonomous driving. (a) Principle of tensor processing—vector coordinates and scalar pixel intensities are extracted and distributed to perform parallel distortion correction and edge detection, followed by capture combination and the subsequent computing tasks, for example, object recognition and decision‐making. (b) Tensor processing of a fisheye capture, and the detailed results of the parallel (c) distortion correction and (d) edge detection tasks for the five regions marked in (b). The results are evaluated in the mean absolute error (MAE) of the curvatures, and the mean contrast of the pixel intensity at the edges. See Figure S23 for the combined output. Heatmaps of the (e) MAE and (f) mean contrast across the fisheye capture before and after tensor processing, respectively. Statistical distribution of the (g) MAE and (h) mean contrast values as estimated, with mean MAE decreased from 2.84 to 0.45 and mean contrast increased from 32.67 to 66.22, proving successful tensor processing. (i) Multiplication‐accumulation operations as required in the conventional computing approaches to perform distortion correction and edge detection tasks for image frames of different resolutions.

Having proved the vector and scalar operation capability of our analog tensor core, we explore its potential to implement large‐scale, single‐layer tensor processing in autonomous driving. We use a public dataset of fisheye capture [30] for the investigation. The dataset includes real images of street views in 1280 × 966 pixels captured by an ultra‐wide fisheye lens. To implement viewfield distortion correction, we design *k*‐matrix (i.e., matrix of *spatial operators*) across the viewfield (Figure S23). To specify, the *spatial operator* at each of the pixels comprises a scale value and the lens configuration constants, where the scale value is determined by the lens configuration and solved by a distortion correction arithmetic [30, 31]. Processing the coordinates across the viewfield using the *k*‐matrix expects to project the distorted coordinates back to the plane. To implement edge detection, *Sobel* edge operators (convolution kernels in 3 × 3 matrix to give a higher resolution [36]) can be used to traverse the pixels in the viewfield. Following our above vector (Figure 3a) and scalar (Figure 4a) operation principles, the MXU of our analog tensor core shall be programmed in blocks to map the *k*‐matrix and *Sobel* edge operators to perform tensor processing. However, given the limited scale of our analog tensor core prototype (Figure 1d), we explore its potential to implement large‐scale tensor processing via simulation, where the *k*‐matrix and *Sobel* edge operators are mapped to a large‐scale MXU.

Figure 5b presents a typical fisheye capture in the dataset and the outputs after the large‐scale tensor processing, where the outputs show successful distortion correction and edge detection. Following this, the outputs are combined to render a viewfield‐corrected edge capture with the edges detected (Figure S24). To exam the tensor processing closely, we show in Figure 5c,d distortion correction and edge detection details at five regions randomly selected and marked in Figure 5b. To quantify the distortion correction, we estimate the curvatures of the buildings (as highlighted in colors) before and after the operation as mean absolute errors (MAEs), and find that the MAEs are substantially decreased at the distorted, edged regions of the viewfield. The MAEs across the viewfield, beyond these five regions, further confirm substantial decrements at the distorted viewfield edges (Figure 5e and Figure S25a,b). The overall mean MAE is decreased from 2.84 to 0.45 (Figure 5g). The results prove an effective viewfield distortion correction. On the other hand, we estimate the mean contrast (Mean Con.) of the pixel intensity at the edges detected in these five regions to evaluate the edge detection. The results show that the mean contrasts are increased significantly. The mean contrast across the viewfield, beyond these five regions, also further confirm significant increments (Figure 5f and Figure S25c,d). The overall mean contrast is increased from 32.67 to 66.22 (Figure 5h). In addition, the overall standard deviation of pixel intensities decreases from 65.32 to 45.68 (Figure S26). The results also prove an effective edge detection. In addition to this fisheye capture, we show in Figure S27 successful viewfield distortion correction and edge detection in four other captures that are taken in the front, left, back, and right omnidirectional viewpoints. As discussed, following tensor processing, the distortion correction and edge detection captures in practical computer vision shall be combined for performing the subsequent object detection and decision‐making tasks. As a demonstration, we prove that the combined captures can be processed using lightweight neural networks that are readily deployed at edge [37] to achieve object detection with high confidence (Figure S28).

The exploration underpins the potential of our analog tensor core in large scales for accelerating computer vision. Using the conventional computing, to perform distortion correction and edge detection, one single pixel can require at least 5 multiplications and 2 accumulations, and 12 multiplications and 11 accumulations, respectively. As such, at least 30 multiplication–accumulation (MAC) operations can be required for one single pixel to implement the above tensor processing. As spatial transformation and edge detection are pixel‐wise computations, processing a frame with 1280 × 966 pixels involves ∼37 M MAC operations. With the rapid advances of the imaging technology, the pixel density is increasing rapidly for a better visual experience and hence, the MAC operations can get exploded using the conventional computing (Figure 5i). Using our analog tensor core in large scales, the enormous amount of visual data can be processed in a high parallelism and output the results in a single clock, that is, saving more than 10^7^ computation operations.

## 3 Conclusion

We have prototyped an analog tensor core using CNT NVMs in conjunction with customized peripheral vector and scalar processing modules. Arising from the linear and symmetric analog weight programming capability of the NVMs, the analog tensor core can be programmed in blocks to distribute analog tensor processing. As demonstration, we use the analog tensor core to perform vector and scalar operations, including spatial transformation and edge detection, and achieve an error of <2.81% in spatial transformation and a signal‐to‐noise ratio of >22 dB in edge detection. Given this tensor processing capability, along with the lightweight and compact design, our analog tensor core shows the potential in accelerating computer vision at edge, for instance, in autonomous driving. As proof‐of‐concept exploration, we perform large‐scale single‐layer tensor processing of street view fisheye captures in autonomous driving with our analog tensor core via simulation, and our results envisage that an analog tensor core in large‐scale can easily facilitate parallel viewfield distortion correction and edge detection of fisheye captures with more than 10^7^ computation operations saved that would otherwise be required in the conventional computing approaches.

Looking ahead to bridging the lab‐to‐fab gap toward real‐world applications, we expect that the fabrication of our tensor core can be adapted to advanced industrial‐scale semiconductor manufacturing processes for the design and manufacture of analog tensor cores in large scales and with high‐level integrations. The manufacture also expects to meet CMOS manufacturing processes toward an integral analog TPU with the analog front end and digital logic fabricated and integrated in one single chip that can be then embedded into the edge visual systems. At the system level, hardware‐algorithm co‐designs are expected and demanded in, for example, developing instructions and error correction codes for practical operation of the analog TPU.

## 4 Methods

### 4.1 Dispersing Semiconducting CNTs

Arc‐discharged single‐walled CNTs (Carbon Solutions Co. Ltd., 1 mg mL^−1^) and poly[n‐(1‐octylnonyl)‐9h‐carbazole‐2,7‐diyl] (PCz, Mackklin, 2 mg mL^−1^) are mixed in toluene by tip sonication (40% × 1 kW, 1 h) and forwarded by a centrifuge process (20 000 g, 2 h) to remove the undispersed bundles and impurities. The supernatant is vacuum filtered with 0.2 µm polytetrafluoroethylene (PTFE) membrane and washed with tetrahydrofuran (THF). The collected residue is redispersed (tip sonication, 40% × 1 kW, 0.5 h) in chloroform to yield a high‐purity semiconducting CNT solution.

### 4.2 NVM Crossbar Array

The NVMs are fabricated on silicon wafer with 300 nm SiO_2_. Before use, the substrate is ultrasonic washed with deionized water, acetone, and isopropanol for 10 min, respectively. The source, drain, gate, and top contact electrodes are all patterned with standard photolithography process. Thermal evaporated titanium (Ti) and gold (Au) are deposited to develop the electrodes − 5/15 nm Ti/Au for the source, drain, and gate electrodes, and 10/200 nm Ti/Au for the top contact electrodes. The CNT thin film is dip coated (lowering with 500 µm s^−1^, lifting with 100 µm s^−1^, repeated for 20 times), baked at 150°C for 1 h, and patterned with photolithography, followed by oxygen plasma (100 sccm, 50 W, 40 s). The dielectric hafnium oxide (HfO_2_, 20 nm) is deposited by atomic layer deposition (ALD). The insulator barrier (SU‐8 2002) is patterned with photolithography and cured on a hotplate with 160°C post‐bake for 30 min.

### 4.3 Electrical Characterization and Testing

The transfer characteristics, multi‐state conductance, and memory retention of the NVMs are measured with Tektronix Keithley 4200A‐SCS equipped with 4225‐RPM pulse modules. The multi‐pulsed potentiation and depression, endurance, and the experimental data processing of the tensor core tests are conducted using Siglent SDG7032A arbitrary waveform generator and SDS2354X digital storage oscilloscope. For this proof‐of‐concept study, the signed weights are realized without a differential‐weight mapping scheme: the programmed CNT NVM conductance defines the weight magnitude, whereas the weight sign is imposed by the polarity of the applied input *V*_ds_ signal. Due to the limitation of the setup, the experimental spatial transformation tests are performed column by column to measure the *x*‐, *y*‐, and *z*‐ output values separately. For the experimental edge detection tests, 4‐channel parallel inputs are used to process the encoded data. See operations of the small‐scale CNT NVM arrays to realize the spatial rotation task (Figure 3a) and the edge detection task (Figure 4a) in Note S6.

### 4.4 Large Scale Tensor Processing

In fisheye capture distortion correction, the relation of a pixel before and after the correction, say (*x_f_*, *y_f_*) and (*x_c_*, *y_c_*) respectively, can be constructed via intermediate 2D‐to‐3D and 3D‐to‐2D projections [30]. For the 2D‐to‐3D projection, the pixel (*x_c_*, *y_c_*) is projected to a 3D coordinate (x, y, z) via the below equation,

$$\left\{\begin{array}{c} \theta =x_{c}\;/f\\ x=fsin\theta \\ z=fcos\theta \\ y=y_{c}\; \end{array}\right.$$

where *f* is the focal length. For the subsequent 3D‐to‐2D projection, the 3D coordinate (*x*, *y*, *z*) is projected to (*x_f_*, *y_f_*) via the below equation,

$$\left\{\begin{array}{c} \theta =\pi /2-arctan\left(z/\sqrt{x^{2}+y^{2}}\right)\\ \rho =k_{1}\;\theta +k_{2}\theta^{2}+k_{3}\theta^{3}+k_{4}\theta^{4}\\ \;x_{f}=\rho x/\sqrt{x^{2}+y^{2}}\\ \;y_{f}=\rho y/\sqrt{x^{2}+y^{2}} \end{array}\right.$$

where the fisheye lens is modelled by ρ  = *k*_1_ θ + *k*_2_θ^2^ + *k*_3_θ^3^ + *k*_4_θ^4^. *k*_1_, *k*_2_, *k*_3_, and *k*_4_ are the distortion coefficients. In large‐scale fisheye capture distortion correction, the CNT MXU acts as the weight matrices to perform the matrix multiplication during the projections, taking the practical hardware performance characteristics into consideration. In fisheye capture edge detection, the task is implemented using the *Sobel* edge detection operators. The object recognition task performed on the distortion correction and edge detection combined results is implemented using the *Ultralytics Yolo11n* model, a lightweight neural network that is readily deployed at edge scenarios [37]. The selection of *YOLO11n* is motivated by its minimal computational overhead (∼2.6 M parameters, ∼6.5 BFLOPs), which aligns with the edge deployment objective of our work. Meanwhile, it does not offset the computational savings achieved by our analog tensor core. More importantly, the results demonstrate that, by offloading the computation‐intensive visual information preprocessing from the neural network to our analog tensor core, a lightweight network model alone is sufficient to achieve reliable object recognition. The code is available on GitHub (https://github.com/ghhucuhk/Analog‐tensor‐processing‐with‐carbon‐nanotube‐in‐memory‐matrix‐multiplications).

## Author Contributions

**Jingfang Pei**, **Lekai Song**, and **Songwei Liu**: methodology, writing – original draft, writing – review and editing, investigation, validation, visualization, formal analysis, conceptualization, data curation. **Guohua Hu**: conceptualization, supervision, funding acquisition, resources, project administration, writing – review and editing, methodology.

## Funding

The authors acknowledge support from RGC (24200521, 15306824), ITC(ITS/150/23FP), CUHK (IDBF25ENG05), SHIAE (RNE‐p3‐21), NSFC (52272160, U2330112), and Sichuan Science and Technology Foundation (2025YFHZ0102, 2024JDRC0021).

## Conflicts of Interest

The authors declare no conflicts of interest.

## Supporting information




**Supporting File 1**: adma74633‐sup‐0001‐SuppMat.docx.

**Supporting File 2**: adma74633‐sup‐0002‐MovieS1.mp4.

**Supporting File 3**: adma74633‐sup‐0003‐MovieS2.mp4.

## Data Availability

The data that support the findings of this study are available from the corresponding authors upon request.
