# Supplementary material for: Analog Tensor Processing With Carbon Nanotube In‐Memory Matrix Multiplications for Edge Computer Vision Acceleration
Source: Adv Mater. 2026 Aug 13;38(57):e74633. doi: 10.1002/adma.74633 (PMC13660814; doi:10.1002/adma.74633)
Supplement: Supplementary file 1 [file ADMA-38-e74633-s001.docx]

Supporting Information

Analog tensor processing with carbon nanotube in-memory matrix multiplications for edge computer vision acceleration

Jingfang Pei, Lekai Song, Songwei Liu, Yingyi Wen, Yaoqiang Zhou, Yang Liu, Pengyu Liu, Wenyu Cui, Xin Lu, Teng Ma, Zegao Wang, and Guohua Hu*

This file contains:

Supplementary Note S1-S6

Figure S1 to S28

Table S1 and S5

Movie S1 and S2

**Supplementary Note S1: Threshold voltage extraction in Figure 2e**

The threshold voltage ($V_{\mathrm{th}}$) of the CNT NVMs is defined and extracted using the *Second Derivative (SD)* method (also known as the *transconductance derivative* method) ^1^. This approach is widely adopted for thin-film transistor operations as it minimizes the ambiguity and subjective errors often associated with visual estimation or linear extrapolation methods.

Specifically, the extraction criteria and procedures implemented in our data analysis algorithm are as follows:

- ***Data smoothing***: To prevent the amplification of experimental electrical noise during numerical differentiation, a moving average filter (convolution window) is first applied to the raw drain-source current ($I_{\mathrm{ds}}$) data.
- ***Derivative calculation***: The first derivative (transconductance, $g_{m}=({dI}_{\mathrm{ds}})/({dV}_{\mathrm{gs}})$) and the subsequent second derivative ($({dI}_{\mathrm{ds}}^{2})/({dV}_{\mathrm{gs}}^{2})$) are sequentially calculated with respect to the gate-source voltage ($V_{\mathrm{gs}}$) data.
- ***Threshold voltage extraction criterion***: The threshold voltage is strictly defined as the gate voltage corresponding to the peak of the second derivative curve within the evaluated sweeping range. Mathematically, it is expressed as: $V_{th}=V_{gs}\left| max({dI}_{\mathrm{ds}}^{2})/({dV}_{\mathrm{gs}}^{2}) \right.$.

**Supplementary Note S2: Noise-limited effective precision during analog programming**

To quantitatively evaluate the analog programming capability of the device, we analyzed the noise-limited effective precision of the conductance evolution during both potentiation and depression. The purpose of this analysis is to estimate how many conductance levels remain distinguishable when the state-to-state fluctuation is explicitly taken into account. The extracted statistics are summarized in Table S1, and the corresponding visualizations are shown in Figure S12.

**1. Statistical definition of programmed conductance states**

For each programming direction, the conductance values measured at a given programming pulse index were grouped as one nominal programmed state. Repeated measurements collected at the same pulse index were then used to construct the conductance distribution of that state. Thus, for the $i$-th programmed state, the measured conductance values are denoted by $\left\{ g_{i,j} \right\}_{j=1}^{M_{i}}$, where $M_{i}$is the number of repeated measurements.

The sample mean and sample standard deviation of state $i$were calculated as

$$\mu_{i}=\frac{1}{M_{i}}\sum_{j=1}^{M_{i}} g_{i,j},$$

$$\sigma_{i}=\sqrt{\frac{1}{M_{i}-1}\sum_{j=1}^{M_{i}} (g_{i,j}-\mu_{i})^{2}}.$$

Here, $\mu_{i}$represents the average conductance of the $i$-th nominal state, while $\sigma_{i}$characterizes its state-to-state fluctuation. To provide a compact parametric description of readout uncertainty, the conductance distribution of each state was approximated by a Gaussian distribution,

$$G_{i}\mathcal{\sim N(}\mu_{i},\sigma_{i}^{2}).$$

This Gaussian approximation was adopted as a practical statistical model for quantifying state overlap and readout error. At the same time, to avoid relying exclusively on this parametric assumption, the experimentally measured distributions were also displayed as empirical cumulative distribution functions (ECDFs) in Figure S12c,d, which provide a model-independent visualization of the overlap among neighboring states.

**2. Metrics for adjacent-state distinguishability**

Because the conductance evolution is monotonic during analog programming, the limiting factor for multi-level resolution is primarily the distinguishability between adjacent programmed states. Therefore, the state resolution was quantified pairwise for neighboring states $i$and $i+1$.

**2.1 Adjacent-state separability**

The separability between two neighboring states was defined as

$$S_{i}=\frac{\mid\mu_{i+1}-\mu_{i}\mid}{\sigma_{i}+\sigma_{i+1}}.$$

This metric measures the spacing between adjacent state means relative to their combined fluctuation width. Larger $S_{i}$indicates better distinguishability, whereas $S_{i}<1$generally implies that the state spacing is comparable to, or smaller than, the combined state broadening.

**2.2 Empirical cumulative distribution function (ECDF)**

For each state, the ECDF was calculated as

$$\hat{F}_{i}(g)=\frac{1}{M_{i}}\sum_{j=1}^{M_{i}} \mathbf{1}(g_{i,j}\leq g),$$

where $1(\cdot)$is the indicator function. The ECDF does not assume any analytical distribution model and was used here as a nonparametric visual cross-check of state overlap. In this work, the ECDF was not used directly to extract the effective bits; rather, it complements the Gaussian-based quantitative analysis by showing how strongly neighboring state distributions interpenetrate in the measured data.

**2.3 Gaussian overlap coefficient**

To quantify the statistical overlap between adjacent states, we computed the Gaussian overlap coefficient

$$\mathrm{OVL}_{i}=\int_{-\infty}^{\infty} \min\left[ f_{i}(g),f_{i+1}(g) \right]\text{ }dg,$$

where $f_{i}(g)$and $f_{i+1}(g)$are the Gaussian probability density functions of states $i$and $i+1$, respectively. The overlap coefficient ranges from 0 to 1. Values near 0 indicate well-separated neighboring states, while values near 1 indicate strong overlap.

**2.4 Pairwise midpoint classification error**

To directly quantify the distinguishability relevant to threshold-based readout, we defined a decision threshold between neighboring states using the midpoint of their means,

$$t_{i}=\frac{\mu_{i}+\mu_{i+1}}{2}.$$

This midpoint threshold was chosen as a simple and symmetric criterion that does not introduce extra fitting parameters and provides an intuitive estimate of state distinguishability under practical readout.

Using this threshold, the probability of misclassifying state $i$as state $i+1$is

$$P(i\to i+1)=1-\Phi\left( \frac{t_{i}-\mu_{i}}{\sigma_{i}} \right),$$

and the reverse misclassification probability is

$$P(i+1\to i)=\Phi\left( \frac{t_{i}-\mu_{i+1}}{\sigma_{i+1}} \right),$$

where $\Phi(\cdot)$is the cumulative distribution function of the standard normal distribution.

The pairwise midpoint classification error was then defined as the average of the two directions:

$$E_{i}=\frac{1}{2}\left[ P(i\to i+1)+P(i+1\to i) \right].$$

This quantity directly estimates the probability that two adjacent conductance states cannot be reliably distinguished using a simple threshold readout.

**3. Definition of effective states and effective bits**

To estimate the available analog precision, we introduced an allowable pairwise-error criterion $\eta$. Two adjacent states were regarded as effectively distinguishable if

$$E_{i}\leq\eta.$$

For a monotonic programming trajectory, an isolated distinguishable state pair does not by itself define a practically usable multi-level analog window. A usable conductance window instead requires a continuous sequence of neighboring states that remain distinguishable along the programming path. Therefore, the effective number of states, $N_{\mathrm{eff}}$, was defined as the length of the longest contiguous state sequence whose adjacent pairs all satisfy $E_{i}\leq\eta$.

Operationally, the adjacent state pairs were first labeled according to whether they satisfy the criterion $E_{i}\leq\eta$. The longest consecutive run of satisfied adjacent pairs was then identified. If this run contains $m$adjacent pairs, the corresponding effective number of states is

$$N_{\mathrm{eff}}=m+1.$$

The corresponding effective number of bits was calculated as

$$B_{\mathrm{eff}}=\log_{2}(N_{\mathrm{eff}}).$$

This definition provides a conservative but practically meaningful estimate of the noise-limited analog precision, because it requires a continuously usable conductance range rather than a few isolated separable state pairs.

To avoid arbitrariness associated with a single threshold, the effective precision was evaluated under three representative criteria:

$$\eta=0.3173,0.1,0.01.$$

Among them, $\eta=0.3173$was included as a relaxed reference level, while $\eta=0.1$and $\eta=0.01$represent increasingly stringent distinguishability requirements.

**4. Extracted results**

The resulting statistics for potentiation and depression are summarized in Table S1, while the corresponding plots are presented in Figure S12. In Figure S12, panels a, c, e, g correspond to potentiation, and panels b, d, f, h correspond to depression. Specifically, panels a,b show the state mean conductance together with the $\pm1\sigma$variation, panels c,d show the ECDFs, panels e,f show the Gaussian overlap coefficient for adjacent states, and panels g,h show the pairwise midpoint classification error.

**4.1 Potentiation**

For potentiation, the average within-state fluctuation was

$$\mathrm{Mean}(\sigma_{G})=3.934563\times{10}^{-1}\pm3.767400\times{10}^{-1}.$$

The adjacent-state separability exhibited a mean of 0.9062, a median of 0.7882, and a minimum of 0.3247. The pairwise midpoint classification error showed a mean of 0.2730, a median of 0.2885, and a maximum of 0.4091. The Gaussian overlap coefficient had a mean of 0.5453, a median of 0.5771, and a maximum of 0.8163.

Using the above error criteria, the extracted effective number of states for potentiation was:

- 72 states, corresponding to 6.170 bits, for $E_{i}\leq0.3173$;
- 7 states, corresponding to 2.807 bits, for $E_{i}\leq0.1$;
- 1 state, corresponding to 0 bits, for $E_{i}\leq0.01$.

**4.2 Depression**

For depression, the average within-state fluctuation was

$$\mathrm{Mean}(\sigma_{G})=3.582391\times{10}^{-1}\pm2.403659\times{10}^{-1}.$$

The adjacent-state separability exhibited a mean of 0.8104, a median of 0.7666, and a minimum of 0.2112. The pairwise midpoint classification error showed a mean of 0.2906, a median of 0.2938, and a maximum of 0.4405. The Gaussian overlap coefficient had a mean of 0.5805, a median of 0.5875, and a maximum of 0.8781.

The extracted effective number of states for depression was:

- 61 states, corresponding to 5.931 bits, for $E_{i}\leq0.3173$;
- 3 states, corresponding to 1.585 bits, for $E_{i}\leq0.1$;
- 1 state, corresponding to 0 bits, for $E_{i}\leq0.01$.

**5. Discussion**

The above results show that the device exhibits a gradual and monotonic analog conductance evolution in both potentiation and depression, indicating the presence of many nominally accessible conductance levels. This trend is visible in the mean conductance trajectories shown in Figure S12a,b.

At the same time, the ECDFs, overlap coefficients, and pairwise midpoint errors consistently indicate substantial overlap between neighboring states. In particular, the mean overlap coefficients are approximately 0.55 for potentiation and 0.58 for depression, while the mean pairwise midpoint errors are approximately 0.27 and 0.29, respectively. These values indicate that adjacent-state confusion remains significant once the conductance fluctuation is taken into account.

As a result, the extracted effective precision is strongly criterion-dependent. Under the relaxed criterion $E_{i}\leq0.3173$, the device supports 72 and 61 effective states for potentiation and depression, respectively, corresponding to about 6.17 and 5.93 bits. However, under the stricter criterion $E_{i}\leq0.1$, the number of robustly distinguishable states decreases to 7 and 3, corresponding to 2.81 and 1.59 bits, respectively. Under the very stringent criterion $E_{i}\leq0.01$, the robust multi-level resolution becomes negligible.

Therefore, this analysis reveals a clear distinction between the nominal analog tunability of the device and its noise-limited robust precision. The device provides many accessible conductance levels in a relaxed sense, but the number of states that remain reliably separable under strict readout-error constraints is considerably smaller. For this reason, the effective bits reported here should be interpreted as a criterion-dependent estimate of analog precision, rather than as a single unique intrinsic device constant.

**Supplementary Note S3: Energy consumption estimation for single device**

When operating our NVMs to process the data, the energy consumption arises from two main processes: i) *Programming*: gate pulses $V_{\mathrm{gs}}$ are applied to program the memory weights; ii) *Data processing*: analog source-drain pulses $V_{\mathrm{ds}}$ are applied to perform multiply-accumulation processing of the data^2^. We now discuss the energy consumption of these two processes:

- ***Programming***

The energy consumption of one single memory weight programming can be estimated by

$E_{\mathrm{programming}}=V_{\mathrm{gs}}\times I_{\mathrm{gs}}\times t_{\mathrm{pulse}}$.

Using our multi-state pulsed memory weight programming method (Figure 2i and Figure S7), the duration of each pulse is set as 500 ns, the maximum operating voltage $V_{\mathrm{gs}}$ is 7.5 V, and the corresponding gate current $I_{\mathrm{gs}}$ is 1.06 × 10^-9^ A (read from the transfer characteristics). Therefore, we estimate the energy consumption for one single weight programming as

$E_{max, programming}=7.5\times1.06\times{10}^{-9}\times500\times{10}^{-9}=3.98\times{10}^{-15} J=3.98\mathrm{fJ}$.

- ***Data processing***

During data processing, no $V_{\mathrm{gs}}$ pulses are applied after the programming, and analog $V_{\mathrm{ds}}$ pulses are applied. To process one single bit of data, the energy consumption can be estimated by

$E_{data processing}=V_{\mathrm{ds}}\times I_{\mathrm{ds}}\times t_{\mathrm{pulse}}$.

In our demonstration (Figure S10-11), the data processing is at a frequency of 1 MHz, the duration of one single bit is 1 μs, the on-state conductance of our NVMs is 30.8 μS (read from the memory weight programming, Figure 2i), and the corresponding current is 3.08 × 10^-5^ A at the maximum input voltage of 1 V. Therefore, we estimate the energy consumption for processing one single bit as

$E_{max, data processing}=1\times3.08\times{10}^{-5}\times1\times{10}^{-6}=3.08\times{10}^{-11} J=30.8\mathrm{pJ}$.

Therefore, based on the above estimations, the energy consumption of our NVMs is ~3.98 fJ for memory weight programming and ~30.8 pJ for data processing.

**Supplementary Note S4: Encoding and decoding in the scalar operation.**

In the workflow of scalar edge detection shown in **Figure 4a**, the encoding stage sequentially converts the grayscale pixel intensities (0-255) into input voltages (0-1 V) for hardware data processing. The decoding stage then converts the measured output voltages back into grayscale values and rearranges them to reconstruct the visual information.

Specifically, the decoding stage includes three steps:

- The output voltage waveform recorded by the oscilloscope is sampled according to the input rate (0.1 ms per data point in this work);
- The sampled voltages are remapped to the grayscale range of 0-255 by normalization, where the minimum and maximum output voltages for each processed image frame are assigned to 0 and 255, respectively;
- The resulting grayscale values are rearranged according to the original processing order to reconstruct the output image frames or video sequence.

In this work, we demonstrate two scalar edge-detection tasks using a 2 × 2 *Roberts* operator: a static full-image case and a 10 × 10 pixel video-stream case. For the static image, the operator scans the image in a raster order (left to right and top to bottom), and the decoded grayscale values are rearranged in the same order to reconstruct the two-dimensional edge image. For the video-stream case, the computation is performed sequentially for each 2 × 2 image patch and then continued to the next patch, so the decoded outputs are rearranged in the same sequence to reconstruct the video stream. Notice that this final reconstruction scheme is part of the data-processing workflow and is implemented on a computer using Python, rather than directly on hardware by the PCB board.

**Supplementary Note S5: Energy consumption estimation for edge detection hardware shown in Figure 4a.**

The scalar convolutional multiply-accumulate operation is implemented using a 4 × 2 CNT NVM array. Specifically, for the *Roberts* operator shown in Figure 4a, the two row-wise scalar operations are

$$V_{in11}\times1+V_{in12}\times0+V_{in13}\times0+V_{in14}\times(-1)$$

and

$$V_{in21}\times0+V_{in22}\times1+V_{in23}\times(-1)+V_{in24}\times0$$

Thus, in one scalar operation, four CNT NVM devices with non-zero weights are active, while the remaining four devices correspond to zero-weight terms. In operation of the CNT NVM devices, as estimated in Supplementary Note 1, the maximum on-state conductance of each CNT NVM device is approximately $G_{\max}=30.8\text{ }\mu S$, and the maximum coded input voltage $V_{in,max}=1V$. Therefore, the maximum power dissipation of one active CNT NVM device can be estimated as

$$P_{\mathrm{device},\max}=G_{\max}V_{\max}=30.8\text{ }\mu S\times(1\text{ }V)^{2}=30.8\text{ }\mu W$$

With the operation duration of

$$t=0.1\text{ }\mathrm{ms}$$

the corresponding maximum energy consumption of one active device is

$$E_{\mathrm{device},\max}=P_{\mathrm{device},\max}t=3.08\text{ }\mathrm{nJ}$$

Since four active devices are involved in one *Roberts* operator scalar operation, the maximum device-level energy consumption of the CNT NVM array is estimated to be

$$E_{\mathrm{array},\max}=4\times3.08\text{ }\mathrm{nJ}=12.32\text{ }\mathrm{nJ}\approx0.0123\text{ }\mu J$$

We note that this array-level device energy is estimated by summing $GV^{2}t$ over the active devices. As positive and negative input voltages are used in our implementation to represent the $"+1"$ and $"-1"$ weights, the output currents from different devices may partially cancel at the summation node. Therefore, the device energy dissipated in the CNT NVM array is not directly equivalent to the net current delivered to the peripheral circuit.

For the estimation of the peripheral-circuit energy consumption, we therefore use the upper-bound net branch current as an independent condition. Since the maximum current contributed by one branch is bounded by

$$I_{in,\max}=G_{\max}V_{\max}=30.8\text{ }\mu A$$

we set

$$I_{in1}=I_{in2}=30.8\text{ }\mu A$$

and the duration of one scalar operation was taken as

$$t=0.1\text{ }ms=1.0\times{10}^{-4}\text{ }s$$

to estimate the energy consumption of the peripheral circuit.

In the following estimation, we consider the dynamic energy consumption in the signal path, including *Joule* dissipation in resistors and conduction loss in forward-biased diodes. The quiescent/static power consumption of the operational amplifiers is not included in this estimation.

To make the analysis clear, the total energy consumption is divided into three parts according to the circuit functionality as shown in Figure S223: (i) $I_{\mathrm{in}}\to node A$, (ii) $Node A\to Node D$, and (iii) $Node D\to V_{\mathrm{out}}$.

***1. Energy consumption of the TIA stage***

The first stage is a standard transimpedance amplifier with a feedback resistor of

$$R_{f1}=100\text{ }k\Omega$$

Thus, the output voltage at node $A$ is

$$V_{out,A}=-I_{\mathrm{in}}R_{f1}=-30.8\times{10}^{-6}\times100\times{10}^{3}=-3.08\text{ }V$$

Under the ideal op-amp approximation, the dominant energy dissipation in this stage is the *Joule* loss on the feedback resistor. The corresponding power is

$$P_{\mathrm{TIA}}=I_{\mathrm{in}}^{2}R_{f1}=(30.8\times{10}^{-6})^{2}\times100\times{10}^{3}\approx94.9\text{ }\mu W$$

Therefore, the energy consumption of one TIA branch during one scalar operation is

$$E_{TIA,1}=P_{\mathrm{TIA}}t=94.9\times{10}^{-6}\times1.0\times{10}^{-4}\approx9.49\times{10}^{-9}\text{ }J\approx9.5\mathrm{nJ}$$

Since the circuit contains two identical branches, the total TIA energy is

$$E_{TIA,total}\approx2\times9.5\text{ }nJ=19\text{ }\mathrm{nJ}$$

***2. Energy consumption of the absolute-value stage***

The second stage is the absolute-value circuit. For the present upper-bound case, the TIA output is

$$V_{out,A}=-3.08\text{ }V$$

According to the diode orientation in the circuit, when $V_{out,A}$ is negative, the branch from $Node A$ to $Node B$ is forward-biased, while the branch from $Node A$ to $Node C$ is cut off. Meanwhile, the feedback branch from $Node D$ to $Node B$ is also forward-biased. Under the ideal op-amp approximation, the non-inverting and inverting inputs satisfy the virtual-short condition, so we take:

$$V_{B}\approx V_{C}\approx0\text{ }V$$

***2.1 Conducting branch from Node A to Node B***

A diode forward voltage drop is approximately of $0.7\text{ }V$, the node immediately after the conducting diode is approximately

$$-3.08+0.7=-2.38\text{ }V$$

Since $V_{B}\approx0\text{ }V$, the current through the $10\text{ }k\Omega$ resistor in this branch is

$$I_{\mathrm{AB}}=\frac{2.38}{1\text{0k}\Omega}=238\text{ }\mu A$$

The power dissipated on this resistor is

$$P_{R_{\mathrm{AB}}}=I_{\mathrm{AB}}^{2}\times10\text{ }k\Omega=(238\times{10}^{-6})^{2}\times10\times{10}^{3}\approx0.566\text{ }\mathrm{mW}$$

The conduction loss on the forward-biased diode is

$$P_{Diode1}=0.7\times238\text{ }\mu A\approx0.167\text{ }\mathrm{mW}$$

***2.2 Feedback branch from Node D to Node B***

In the feedback path, the current is approximately equal to the conducting-branch current under current balance at the virtual-ground node, i.e.

$$I_{\mathrm{DB}}\approx I_{\mathrm{AB}}\approx238\text{ }\mu A$$

Thus, the resistor dissipation in the feedback branch is

$$P_{R_{f2}}=I_{\mathrm{DB}}^{2}\times10\text{ }k\Omega\approx0.566\text{ }\mathrm{mW}$$

and the diode conduction loss is

$$P_{Diode3}=0.7\times238\text{ }\mu A\approx0.167\text{ }\mathrm{mW}$$

The output voltage of $Node D$, $V_{out,D}$ can also be estimated from this branch. Since the branch contains one forward-biased diode followed by a $10\text{ }k\Omega$ resistor connected to the virtual-ground node,

$$\frac{V_{out,D}-0.7}{10\text{ }k\Omega}\approx238\text{ }\mu A$$

and therefore

$$V_{out,D}\approx3.08\text{ }V$$

***2.3 Grounded resistor at Node D***

The output $Node D$ is also connected to ground through a $10\text{ }k\Omega$ resistor. Therefore, the current through this resistor is

$$I_{D,GND}=\frac{V_{out,D}}{R}=\frac{3.08}{10\text{ }k\Omega}=308\text{ }\mu A$$

and the corresponding power dissipation is

$$P_{R_{D,GND}}=\frac{V_{out,D}^{2}}{R}=\frac{{3.08}^{2}}{10\text{ }k\Omega}\approx0.949\text{ }\mathrm{mW}$$

***2.4 Total energy of one absolute-value circuit***

Summing the main contributions, the total power consumption of one absolute-value circuit is estimated as

$$P_{\mathrm{abs},1}\approx0.566+0.167+0.566+0.167+0.949=2.415\text{ }\mathrm{mW}$$

Thus, the energy consumption of one absolute-value circuit during one scalar operation is

$$E_{\mathrm{abs},1}=P_{\mathrm{abs},1}t=2.415\times{10}^{-3}\times1.0\times{10}^{-4}=2.415\times{10}^{-7}\text{ }J\approx0.242\text{ }\mu J$$

Since there are two identical absolute-value circuits, the total energy consumption of this stage is

$$E_{\mathrm{abs},\mathrm{total}}\approx2\times0.242\text{ }\mu J=0.484\text{ }\mu J$$

***3. Energy consumption of the summation stage***

The third stage is an inverting summation amplifier. The outputs $Node D$ of the two absolute-value circuits are each connected to the summation $Node E$ through a $10\text{ }k\Omega$input resistor. The non-inverting input is grounded, so under the ideal op-amp approximation,

$$V_{in,E}\approx0\text{ }V$$

Since each branch has

$$V_{out,D}\approx3.08\text{ }V$$

the current through each $10\text{ }k\Omega$ input resistor is

$$I_{in,E}=\frac{3.08}{10\text{ }k\Omega}=308\text{ }\mu A$$

The two branches therefore provide a total current of

$$I_{\mathrm{sum}}=2\times308\text{ }\mu A=616\text{ }\mu A$$

With a feedback resistor of $R_{f3}=10\text{ }k\Omega$, the output voltage is

$$V_{\mathrm{out}}=-I_{\mathrm{sum}}\times10\text{ }k\Omega=-6.16\text{ }V$$

***3.1 Input resistor dissipation***

The power dissipated on each input resistor is

$$P_{R_{in}}=\frac{{3.08}^{2}}{10\text{ }k\Omega}\approx0.949\text{ }\mathrm{mW}$$

Thus, the two input resistors together dissipate

$$P_{R_{in,\mathrm{total}}}=2\times0.949=1.898\text{ }\mathrm{mW}$$

***3.2 Feedback resistor dissipation***

The voltage drop across the feedback resistor is approximately $6.16\text{ }V$, so the power dissipated on the feedback resistor is

$$P_{R_{f3}}=\frac{{6.16}^{2}}{10\text{ }k\Omega}\approx3.795\text{ }\mathrm{mW}$$

***3.3 Total energy of the summation stage***

The total power of the summation stage is therefore

$$P_{\mathrm{sum}}=1.898+3.795=5.693\text{ }\mathrm{mW}$$

and the corresponding energy for one scalar operation is

$$E_{\mathrm{sum}}=P_{\mathrm{sum}}t=5.693\times{10}^{-3}\times1.0\times{10}^{-4}=5.693\times{10}^{-7}\text{ }J\approx0.569\text{ }\mu J$$

***4. Total estimated energy consumption for one scalar operation***

The total signal-path energy consumption is the sum of the above three parts:

$$E_{\mathrm{total}}=E_{TIA,total}+E_{\mathrm{abs},\mathrm{total}}+E_{\mathrm{sum}}$$

Substituting the calculated values yields

$$E_{\mathrm{total}}=0.019\text{ }\mu J+0.484\text{ }\mu J+0.569\text{ }\mu J\approx1.072\text{ }\mu J$$

Therefore, the total energy consumption of the peripheral circuit in Figure 4a for one scalar edge-detection operation is estimated to be

$$E_{\mathrm{total}}=1.072\mu J$$

Therefore, under the upper-bound condition of $I_{in1}=I_{in2}=30.8\text{ }\mu A$ and an operation duration of $0.1\text{ }\mathrm{ms}$, the signal-path energy consumption of the peripheral circuit in Figure 4a is estimated to be approximately $1.07\text{ }\mu J$ per scalar edge-detection operation. In this estimation, as we present in Table S5, CNT NVMs consume a minimal energy, while among the three functional blocks, the TIA stage contributes only a small fraction. The major energy consumption arises from the absolute-value stage and the final summation stage.

**Supplementary Note S6: Hardware operations to realize the spatial rotation task (Figure 3a) and the edge detection task (Figure 4a).**

In this work, due to the limited number of parallel input channels available in our current laboratory setup, the demonstrations in this work are performed in a row-by-row (or column-by-column) manner, rather than a fully parallel large-scale matrix operation. In fact, indeed, the input pixels in the edge-detection demonstration are processed sequentially using a small number of programmed kernels. Specifically, as shown in Figure 4a, our experiment physically employed only two 4 × 1 CNT NVM arrays, which are programmed to implement the two 2 × 2 kernels of the *Roberts* operator. These two kernels are then repeatedly used to process different pixel windows across the full image/video stream in a sequential manner. As such, the edge-detection experiment does not really rely on a large number of simultaneously programmed kernels distributed over the entire frame. In this sense, the experiment provides an experimental hardware validation of visual signal processing using CNT NVMs.

In this context, to prototype the spatial rotation task (Figure 3a) and the edge detection task (Figure 4a), we use the small-scale CNT NVM arrays following the below operations:

- ***For the spatial rotation task (Figure 3a):*** for one rotation, 867 data points are processed. For each point, one multiplication is required along each of the three axes ($x$, $y$, and $z$). Therefore, the total number of multiply-accumulate operations is:

$$867\times3=2601$$

This task uses 3 active CNT NVM devices, and each device processes 867 data points. In our demonstration, each operation takes 0.1 ms, the total execution time is:

$$867\times0.1 \text{ms}=86.7 \text{ms}$$

As a hypothesis, if one complete spatial rotation of the 3D model is to be executed fully in parallel within 0.1 ms, 867 arrays of 3 × 3 CNT NVMs are required. Since each 3 × 3 array contains 9 devices, the total number of devices is:

$$867\times9=7803$$

In summary, for one spatial rotation task, 3 active CNT NVMs are used in the current sequential implementation, with a total of 2601 multiply-accumulate operations and a total execution time of 0.0867 s. Under large-scale fabrication, if 867 $3\times3$arrays, corresponding to 7803 devices, are operated in parallel, the entire task can be completed in a single operation step.

- ***For the edge detection task (Figure 4a)****:* For a 640 × 320 image, two 2 × 2 kernels are used for edge detection in the $x$- and $y$-directions, respectively. As described in the manuscript, these correspond to two 4 ×1 CNT NVM arrays. Each kernel scans the image over:

$$639\times359=229401$$

positions. Since each kernel contains 2 active devices, the total number of multiply-accumulate operations for one kernel is:

$$639\times359\times2=458802$$

Because two kernels are used, the total number of multiply-accumulate operations becomes:

$$639\times359\times2\times2=917604$$

Accordingly, the total execution time is:

$$917604\times0.1\text{ ms}=91760.4\text{ ms}$$

If the edge detection of one image is to be completed fully in parallel within $0.1$ms, $639\times359\times2=458802$arrays of $4\times1$devices are required. Since each $4\times1$array contains 4 devices, the total number of devices is:

$$458802\times4=1835208$$

For one complete edge detection task on a 640 × 360 image, 4 active devices are used in the current implementation, with a total of 917,604 multiply-accumulate operations and a total execution time of 91.7604 s. Under large-scale fabrication, if 458,802 4 × 1 arrays, corresponding to 1,835,208 devices, are operated in parallel, the entire image-processing task can be completed in a single operation step.

For video-stream edge detection, we processed 100 frames with a frame size of $10\times10$. According to the present setup, the required number of multiply-accumulate operations is:

$$9\times9\times50\times2\times2=16200$$

Thus, the corresponding execution time is:

$$16200\times0.1\text{ }\text{ms}=1620\text{ }\text{ms}$$

To achieve temporal parallel processing for a $10\times10$frame, $9\times9\times2=162$arrays of $4\times1$devices are required. Since each array contains 4 devices, the total number of devices is:

$$162\times4=648$$

For one complete video-stream edge detection task under the present setup, 4 active devices are used sequentially, with a total of 16,200 multiply-accumulate operations and a total execution time of 1.62 s. Under large-scale fabrication, if 162 4 × 1 arrays, corresponding to 648 devices, are operated in parallel, the **one-frame** task can be completed in a single operation step.

**Supplementary Figures**

**Figure S1. Peripheral vector and scalar processing modules.** (a) The printed circuit board (PCB) with CNT NVM chip wire bonded with the peripheral circuits (replotted from Figure 1d). (b) Schematic circuit of the analog tensor core, where a 3 × 3 CNT NVM array is connected with 3 TIAs for spatial transform readout (also shown in Figure 3a), and a 4 × 2 CNT NVM array is connected with a circuit group (TIA, absolute and summation) for edge detection readout (also shown in Figure 4a). The source, drain, and gate voltages are supplied from arbitrary waveform generators through a designed pin-header interface and wired connections, while the output signals are acquired using an oscilloscope.

**Figure S2. Programming setup of the CNT NVM array.** (a) PCB board integrating the wire-bonded CNT NVM. Dedicated pin-header interfaces are provided for the source, drain, and gate terminals, allowing external voltage inputs from waveform generators through wired connections. (b) Circuit diagram illustrating the programming and operation method used in this work. For a selected row of devices, programming voltage pulses are applied to the gate terminals to set the device states. After programming, input signals are applied through $V_{\mathrm{ds}}$ for data processing.

**Figure S3. UV-Vis-NIR absorption spectra of the CNT and sorting polymer PCz solutions.** (a) The S_33_ and S_22_ peaks prove successful sorting of the semiconducting single-walled CNTs by the sorting polymer PCz^3^.

**Figure S4. CNT thin film deposition.** (a) CNT film deposition on 4-inch SiO_2_/Si wafer by dip coating of the PCz sorted CNT solution. (b) Scanning electron microscopic (SEM) image of the CNT film on SiO_2_/Si wafer, showing uniform CNT film with high density. Scale bar – 1 μm.

**Figure S5. Microscopic characterization of the CNT NVMs.** (a) Schematic structure of the CNT NVM devices. (b) Optical microscopic image of a typical NVM device in crossbar array, and (c) atomic force microscopic (AFM) image of the CNT channel. Scale bars – (b) 100 μm, (c) 200 nm.

**Figure S6. Long-term memory retention of the CNT NVMs under pulsed gate voltage signals.** Long-term memory retention of a typical NVM device under one single gate pulse $V_{\mathrm{gs}}$ in (a-c) negative amplitudes and (d-f) positive amplitudes. The gate pulse $V_{\mathrm{gs}}$ settings are shown in (b) and (e). The drain-source pulses $V_{ds}$ are set as 0.5 V DC in all the tests. (a, d) The retention behavior during the initial 0 to 20 s. (c, f) The stabilized retention behavior during the final 190 to 200 s. The tests prove a long-term memory retention of our CNT NVMs.

**Figure S7. Conductance modulation under continuous constant-amplitude pulse train stimulation.** Weight programming of a CNT NVM device under 10 repeated cycles of potentiation and depression. 30 positive pulses (amplitude 3 V, duration 10 μs, interval 990 μs) followed by 30 negative pulses (amplitude -3V, duration 10 μs, interval 990 μs) are applied to the gate during one cycle of the potentiation and depression test. The drain-source voltage $V_{\mathrm{ds}}$ is 0.5 V DC. The red dots correspond to the conductance of potentiation, and the blue dots correspond to the conductance of depression. The nonlinearity factor is calculated as 3.96 and -11.80 for the potentiation and depression stage, respectively, showing a limited linearity under pulse train stimulation.

**Figure S8. Pulse train of gate voltage signal** $\boldsymbol{V}_{\mathbf{gs}}$ **to program the memory weight of the CNT NVMs.** (a) Schematic test setup of CNT NVM conductance state modulation, and (b) schematic programming and reading stage of the test. (c) Pulse train $V_{\mathrm{gs}}$, and (d) the detailed pulsed signal parameter and the memory weight read operation. The drain-source $V_{\mathrm{ds}}$ is set as 0.5 V DC. The pulse train $V_{\mathrm{gs}}$ enables linearity and symmetry in the potentiation and depression operations of our CNT NVMs. Note that the pulse amplitudes used in the linearized multi-pulse programming (+3.5 V to +7.5 V, and -0,5 V to -4.5 V) are selected to optimize the linearity and symmetry, rather than to maximize the programming window; these values are still within the device operating range and below the maximum available programming amplitude of +/- 8 V.

**Figure S9. Time-dependent modulation of device conductance.** The conductance state of a typical CNT NVM device is programmed using single gate pulses with an amplitude of 8 V in (a) and −8 V in (b), while varying the pulse duration. Before each programming event, a long pulse (0.1 s) with the opposite polarity (−8 V for (a) and 8 V for (b)) is applied to reset the device to its initial state. During the measurement, a $V_{\mathrm{ds}}$ of 0.5 V is used to read out the current and extract the conductance. (a) shows the programming process from a low-conductance state to a high-conductance state, whereas (b) shows the modulation from a high-conductance state to a low-conductance state. This shows that a longer pulse duration at the programming amplitude of +/- 8 V can provide a wider weight programming window when needed.

**Figure S10. Response of the CNT NVMs.** (a) 10-cycle response of a typical NVM device under drain-source pulses $V_{\mathrm{ds}}$ of 0.2 V amplitude at a frequency of 1 MHz, and (b) the detailed rising edge of the output, showing the response delay and switching time are ~23 ns and ~53 ns, respectively, proving a high data processing speed of our CNT NVMs. During the test, the NVM is programmed at the initial memory weight state.

**Figure S11. Endurance test of the CNT NVMs.** A train of alternatively switching paired pulsed source-drain voltage signal $V_{\mathrm{ds}}$ of +/- 0.5 V is applied to the drain terminal. The pulsed signal frequency is 1MHz, corresponding to a data processing speed of 1 Mbit/s. During the test, the device is programmed at the initial memory weight state. The device undergoes 10^7^ consecutive pulsed cycles. The current outputs $I_{\mathrm{ds}}$ in positive and negative amplitudes to the paired pulsed $V_{\mathrm{ds}}$ signals in each of the cycles are shown as the blue and green dots, respectively, and the corresponding conductance are shown as the red and orange dots, respectively. The stable memory (i.e. the conductance) behavior under the alternatively switching paired positive and negative pulsed $V_{\mathrm{ds}}$ signals proves that our CNT NVMs can undergo stable data processing without shifting the memory weight. Here, the test should be more precisely regarded as a read stability/read endurance test, in which small drain-input pulses are applied to probe the stability of a programmed conductance state under repeated signal-input operation, rather than to induce deliberate ON/OFF switching. This measurement therefore evaluates the robustness of stored analog weights under application-relevant read conditions, whereas the endurance of repeated conductance reprogramming is reflected by Figures 2j–k.

**Figure S12. Statistical analysis of noise-limited effective precision during analog programming**. Panels (a, c, e, g) correspond to potentiation, and panels (b, d, f, h) correspond to depression. (a, b) Mean conductance of each programmed state with ±1σ error bars, showing the gradual analog conductance evolution and the state-to-state fluctuation. (c, d) Empirical cumulative distribution functions (ECDFs) of the measured conductance values for all programmed states, providing a model-independent visualization of the overlap among neighboring states. (e, f) Gaussian overlap coefficient between adjacent conductance states, quantifying the degree of statistical overlap between neighboring state distributions. (g, h) Pairwise midpoint classification error between adjacent states, calculated using the midpoint between neighboring state means as the decision threshold. These results are used to determine the effective number of states and the corresponding effective number of bits under different allowable error criteria.

**Figure S13. Experimental inputs and outputs of spatial transformation of the 3D model shown in Figure 3c.** (a) x-, y-, and z- input signals of a total of 837 coordinates of the 3D model. (b) Output signals to the coordinates after the identical spatial transformation operation (i.e. 0^o^ rotation around the x- y- and z-axes). The nearly identical output waveforms with input in (a) proves successful spatial data processing. (c) Output signals to the coordinates after 90^o^ rotation operation around the x-axis. The amplitudes in (a) correspond to the coordinate values of the model. The amplitudes in (b) and (c) are output voltages corresponding to the coordinate values after spatial transformation. The output voltages are sampled according to the input frequency intervals, and are then normalized and reconstructed to output the coordinate values. The gray regions in (a), (b) and (c) correspond to the regions presented in Figure 3c (1-3), respectively.

**Figure S14. Experimental data points plotted in space coordinate system of the 3D model shown in Figure 3d.** (a) The input coordinates and (b) output coordinates after the identical spatial transformation operation. The outputs with 90^o^, 180^o^ and 270^o^ rotation operations around (c-e) x-, (f-h) y-, and (i-k) z- axes, respectively. See Movie S1 for the spatial transformation.

**Figure S15. Distribution of pixel intensity in Figure 4b.** The distribution of pixel intensity in (a) input frame 1 and (b) frame 99, and in the edge detected (c) output frame and (d) frame 99. The distributions of the input frames in (a) and (b) show broader intensity spectra, with the intensities distributed relatively evenly. After edge detection, most non-edge areas are assigned a value of “0”, while a small number of pixels at the edges are assigned “1”, as shown in Figure 4a. This process compresses the distribution spectra and pushes for stronger pixel intensities, as shown in (c) and (d). In addition, as demonstrated, the standard deviation of the pixel intensity decreases significantly, proving the effectiveness of the edge detection using our tensor core. Note that, due to the design of our back-end circuits, the output readout voltages are negative and as such, the reconstructed images show inverted intensity: the edges appear dark and the non-edge regions bright, opposite to the typical algorithm output. This inversion does not affect the effectiveness of edge detection.

**Figure S16. Experimental edge detection output heatmaps of frame 99 shown in Figure 4c.** An area of 10 × 10 pixels is used to evaluate the (a) vertical and (b) horizontal edge detection from 11 × 11 pixels input using our analog tensor core. The values presented in the heatmaps are the output voltages ($V_{\mathrm{out}}$) from the tensor core, and the edges detected are enclosed by the red outlines. To evaluate the detection performance quantitatively, we estimate the signal-to-noise ratio (SNR) using

$SNR (dB)=20{log}_{10}(\frac{\mu_{\mathrm{edge}}-\mu_{\mathrm{background}}}{\sigma_{\mathrm{background}}})$,

where $\mu_{\mathrm{edge}}$ is the averaged $V_{\mathrm{out}}$ in the edge region, while $\mu_{\mathrm{background}}$is the averaged $V_{\mathrm{out}}$ in the non-edge region, and $\sigma_{\mathrm{background}}$ is the standard deviation (s. d.) of the $V_{\mathrm{out}}$ in the non-edge region. The SNR is estimated as 22.69 dB for region (a) and 22.64 dB for region (b), respectively. In addition, dynamic range is also used to evaluate the edge detection,

$Dynamic range \left( \mathrm{dB} \right)=20{log}_{10}(\frac{V_{\max}}{V_{\min}})$,

where $V_{\max}$ is the maximum $V_{\mathrm{out}}$ in the whole region, while $V_{\min}$ is the minimum $V_{\mathrm{out}}$. The dynamic range is estimated as 30.13 dB for (a) and 27.85 dB for (b), respectively. The SNR and dynamic range, approaching the industrial application standard, prove the ability of our analog tensor core in performing efficient, low-pression edge detection scenarios at edge.

**Figure S17. Experimental input and output comparison in edge detection of frame 99 shown in Figure 4c.** (a-b) The input frame. (c-d) The output frame after the edge detection operation using our analog tensor core. An area of 11 × 11 pixels of the input frame is used for the comparison, and the corresponding output frame is presented in 10 × 10 pixels. The values presented are the grayscale values of each pixel, i.e. pixel intensity, $I_{n}$. The red lines in (c-d) enclose the vertical and horizontal edges detected in the output frame, respectively, and those in (a-b) mark the corresponding edges in the vertical and horizontal direction the input frame, respectively. Mean contrast in pixel intensity is introduced to evaluate the edge detection,

$Contrast=\frac{1}{N}\sum_{n=1}^{N} (I_{n, left}-I_{n, right})$for vertical edge detection,

or alternatively, $Contrast=\frac{1}{N}\sum_{n=1}^{N} (I_{n, up}-I_{n, bottom})$for horizontal edge detection,

where the differences in $I_{n}$ of each pixel on both sides of the edges are averaged and evaluated. A larger mean contrast means that the edges are more determined from the neighboring pixels. As estimated, the mean contrast of the input frame is 81.27 for (a) and 55.18 for (b), while the mean contrast of the corresponding output frame is 116.17 for (c) and 80.95 for (d). The substantial increments in the mean contrast of (c-d) indicate that the edges are well detected by our analog tensor core.

**Figure S18. Visualized experimental edge detection of the video flow shown in Figure 4e.** (a) Input and (b) the reconstructed edge detection output of the video flow frame 86 to 135. The edges are clearly detected when the object passes during frame 105 to 109. See Movie S2 for the edge detection.

**Figure S19. Experimental real-time edge detection output heatmaps of frame 105 (a) and frame 108 (b) shown in Figure 4e.** An area of 9 × 9 pixels is used to evaluate the real-time edge detection from 10 × 10 pixels input using our analog tensor core. The values presented in the heatmaps are the output voltages ($V_{\mathrm{out}}$) from the tensor core, and the edges detected are enclosed by the red outlines. The SNR is estimated as 10.76 dB for frame 105 (a) and 13.55 dB for frame 108 (b), respectively. The dynamic range is estimated as 22.78 dB for frame 105 (a) and 22.47 dB for frame 108 (b), respectively. This proves the ability of our analog tensor core in performing efficient, low-pression real-time edge detection scenarios at edge.

**Figure S20. Experimental input and output comparison in real-time edge detection of frame 105 and frame 108 shown in Figure 4e.** (a-b) The input frames. (c-d) The output frames after real-time edge detection operation using our analog tensor core. An area of 10 × 10 pixels of the input frames is used for the comparison, and the corresponding output frames are presented in 9 × 9 pixels. The values presented are the grayscale values of each pixel, i.e. pixel intensity, $I_{n}$. The red lines in (c-d) enclose the edges detected in the output frames, respectively, and those in (a-b) mark the corresponding edges in the input frames, respectively. As estimated, the mean contrast of the input frames is 86.5 for frame 105 (a) and 69.6 for frame 108 (b), while the mean contrast of the corresponding output frame is 149.56 for frame 105 (c) and 160.44 for frame 108 (d). The substantial increments in the mean contrast of (c-d) indicate that the edges are well detected by our analog tensor core in real-time.

**Figure S21. Experimental edge detection outputs of 9 selected pixels in the video flow shown in Figure 4f.** The peaks represent the detected edges of the corresponding pixels in the video flow. The coordinates represent the selected pixels. The results here show the first 50 frames of the video flow. Each frame is processed in a time scale of 0.1 ms. The output voltages are sampled according to the input frequency intervals, and are then normalized and reconstructed to output the pixel intensity values. The gray region corresponds to the regions presented in Figure 4f.

**Figure S22.** **Circuit diagram of the peripheral circuit for scalar edge detection task** shown in Figure 4a.

**Figure S23. Visualized *k*-matrix (i.e. matrix of the spatial operators) of the viewfield distortion correction task shown in Figure 5a.** (a) Mapping of the scale values of the *k*-matrix across the viewfield. (b) The general formular of the *k*-matrix, where the scale value variates at the specific pixel across the viewfield, and the $C_{x}$ and $C_{y}$ are the constants corresponding to the fisheye lens configuration. Across the *k*-matrix, the scale value is solved by the distortion correction arithmetic as reported in ref. ^4^. $C_{x}$ and $C_{y}$ are 4 and -3, respectively, according to the configuration of the fisheye lens. (c) Four specific *k*-matrix units (i.e. spatial operators) at four specific locations across the viewfield. The four locations are shown in (a).

**Figure S24. Tensor processing workflow using our CNT tensor core.** The input image as shown in (a) is processed in (b) parallel tensor processing to perform the distortion and edge detection tasks. The images after the parallel distortion and edge detection operations are then combined to render the viewfield-corrected edge detection image shown in (c). The combined result is cropped at the edges for the convenience of viewfield-correction representation.

**Figure S25. Selected areas for quantifying the parallel tensor processing shown in Figure 5e and f.** The 36 areas of the (a) fisheye capture are marked with yellow lines for quantifying the (b) distortion correction and (c) edge detection, respectively. The corresponding results are marked with red lines for distortion correction in (b), and grayscale lines for edge detection in (d). To quantify the distortion correction, we select the regions that are expected to be straight lines in the real world. For each region, we apply the least squares method to fit the set of points before and after correction, and then measure its deviation from the ideal straight line. The deviation is quantified as the mean absolute error (MAE), defined as the mean of the perpendicular distances from each point to the fitted line:

$d_{i}=\frac{\left| {kx}_{i}-y_{i}+b \right|}{\sqrt{k^{2}+1}}$*,*

$MAE=\frac{1}{N}\sum_{i=1}^{N} d_{i}$,

where $\left( x_{i},y_{i} \right)$ represents the coordinates of each point, and $k$ and $b$ are the slope and intercept of the fitted line, respectively. By comparing the MAE values before and after correction, we can quantitatively assess the improvement in the straightness and thus, the effectiveness of the distortion correction. The mean MAE of the 36 areas decreases from 2.84 to 0.45 (Figure 5g), proving an efficient distortion correction. To quantify the edge detection, we select the regions with edges, and calculate the mean contrast via the method described in Figure S17. Note that, to minimize the impact of the inaccurate manual line placement and complex surrounding edges on the accuracy of the quantitative results, we calculate the edge strength as the mean intensity across a three-pixel-wide line centered on the selected edge segment (i.e., averaging one pixel on each side of the edge). For the non-edge regions, we average the intensities of five pixels on each side of the edge segment. This approach enables a more accurate measurement of mean contrast across the edge. The effectiveness of edge detection can then be quantitatively assessed by the observed increase in contrast (from 32.67 to 66.22) after edge detection.

**Figure S26.** **Distribution of pixel intensity in Figure 5b.** The distribution of pixel intensity in (a) the fisheye capture input and (b) the edge detected output. The standard deviation of the pixel intensity decreases significantly from 65.32 to 45.68, proving the effectiveness of the edge detection using our tensor core.

**Figure S27. Large-scale image processing in autonomous driving using our CNT analog tensor core via simulation.** (a) Schematic omnidirectional perception in autonomous driving. The front, left, back, and right sides of visual information are captured from the corresponding fisheye cameras. (b) Omnidirectional visual information perception, accomplishing parallel distortion correction and edge detection tasks using our analog tensor core. The outputs from distortion correction and edge detection are subsequently combined to output the viewfield-corrected edge detection images. Note that, as the parameters of the fisheye cameras are different in the front, left, back, and right four sides, the *k*-matrixes in each side are adjusted accordingly in the distortion correction task^5^.

**Figure S28. Object recognition after the large-scale tensor processing using our CNT analog tensor core.** The object recognition results in the (a) front, (b) left, (c) back, and (d) right view of the street after the distortion correction and edge detection tensor processing. The cars and bicycles are accurately recognized with high confidence, proving the effectiveness of performing tensor processing using our analog tensor core.

**Supplementary Tables**

**Table S1.** **Statistical noise-limited effective precisions during analog programming for potentiation and depression**. For each programming direction, the table summarizes the mean within-state conductance fluctuation mean, adjacent-state separability, pairwise midpoint classification error, Gaussian overlap coefficient, and the extracted effective number of states and effective number of bits under different allowable pairwise-error criteria ($E_{i}\leq0.3173$, $0.1$, and $0.01$). The effective number of bits is calculated as $B_{\mathrm{eff}}=\log_{2}(N_{\mathrm{eff}})$.

|  | | *Potentiation* | *Depression* |
| --- | --- | --- | --- |
| Mean | | 0.3935 | 0.3582 |
| Standard deviation | | 0.3767 | 0.2404 |
| Adjacent separability | Mean | 0.9062 | 0.8104 |
|  | Median | 0.7882 | 0.7666 |
|  | Min | 0.3247 | 0.2112 |
| Pairwise midpoint error | Mean | 0.2730 | 0.2906 |
|  | median | 0.2885 | 0.2938 |
|  | Max | 0.4091 | 0.4405 |
| Gaussian overlap coefficient | Mean | 0.5453 | 0.5805 |
|  | Median | 0.5771 | 0.5875 |
|  | Max | 0.8163 | 0.8781 |
| At pair-error ≤ 0.3173 | Effective states | 72 | 61 |
|  | Effective bits | 6.170 | 5.931 |
| At pair-error ≤ 0.1 | Effective states | 7 | 3 |
|  | Effective bits | 2.807 | 1.585 |
| At pair-error ≤ 0.01 | Effective states | 1 | 1 |
|  | Effective bits | 0.000 | 0.000 |

**Table S2. Quantitative comparison of representative programming/switching-related energy in CIM-related emerging memory devices.** To benchmark the energy efficiency of the CNT NVM, the table compares the estimated single-device programming energy in this work with representative programming/switching-related energy values reported for CIM-related emerging memory devices, including RRAM, PCM, and FeFET technologies. Because operation definitions and test conditions vary across the literature (for example, switching, SET/RESET, write/erase, weight update, or synaptic event), the comparison is intended as an order-of-magnitude quantitative benchmark rather than a strict one-to-one evaluation. Although the present device operates through a charge-trapping mechanism, directly comparable single-operation energy data for charge-storage-based computing memories remain relatively limited and non-uniformly reported; therefore, the main comparison is made against the most widely adopted CIM memory platforms.

| *Technology* | *Energy consumption*  *(single device, single operation)* | *Energy type* | *Reference* |
| --- | --- | --- | --- |
| **Charge trap** | **3.98 fJ** | **Weight programming** | **This work** |
| RRAM | Few-fJ level | Switching | Ref. 6 |
| RRAM | 100 fJ | Switching | Ref. 7 |
| RRAM | 0.82 pJ | Switching | Ref. 8 |
| PCM | <1.5 pJ | Weight programming | Ref. 9 |
| PCM | 3.16 pJ | Weight programming | Ref. 10 |
| FeFET | 40-234 fJ | Weight programming | Ref. 11 |
| FeFET | 2.0 fJ | Weight programming | Ref. 12 |
| FeFET | 0.36 fJ | Weight programming | Ref. 13 |
| FeFET | 0.0003 fJ | Weight programming | Ref. 14 |

**Table S3. Error evaluation of spatial transformation in Fig. 3b-c, (2).** To quantitively evaluate the identical spatial transformation (i.e. 0^o^ rotation around the x- y- and z-axes) of the 5 selected points in Fig. 3b-c, (2), we present in the table the coordinates of the inputs and outputs, respectively, and the estimated percentage error (PE) and relative *Euclidean* error (REE). PE is estimated by $PE=\left| c_{\mathrm{output}}-c_{\mathrm{input}} \right|/\left| c_{\mathrm{input}} \right|\times100\%$, where $c$ represents the coordinates of the inputs and outputs along the x- y- and z-axes. RPE is estimated by $REE=D{/A}_{\mathrm{input}}\times100\%$, where $A_{input}$ is the magnitude of the coordinate vector, and $D$ is the *Euclidean* distance, $D=\sqrt{\left( x_{output}-x_{input} \right)^{2}+\left( y_{output}-y_{input} \right)^{2}{+\left( z_{output}-z_{input} \right)}^{2}}$. PE evaluates the spatial transformation error at an individual coordinate along the x- y- and z-axes, and REE evaluates the overall error of the spatial transformation. As estimated, PE is <0.73% and REE is <1.12% for the 5 selected points. PE and REE for the 867 coordinates of the 3D model are <1.47% and <2.81%, respectively, proving the high accuracy spatial transformation performed using our CNT tensor core.

| *Points* | *Input* | | | *Output* | | | *PE* | | | *REE* |
| --- | --- | --- | --- | --- | --- | --- | --- | --- | --- | --- |
|  | x | y | z | x’ | y’ | z’ | x-axis | y-axis | z-axis | – |
| 1 | 0.160 | 0.700 | 0.715 | 0.163 | 0.696 | 0.713 | 0.42% | 0.42% | 0.15% | 0.61% |
| 2 | 0.097 | 0.786 | 0.181 | 0.096 | 0.781 | 0.188 | 0.04% | 0.55% | 0.73% | 1.12% |
| 3 | 0.198 | 0.948 | 0.442 | 0.200 | 0.944 | 0.442 | 0.14% | 0.40% | 0.00% | 0.40% |
| 4 | 0.440 | 0.284 | 0.627 | 0.437 | 0.278 | 0.623 | 0.23% | 0.52% | 0.41% | 0.87% |
| 5 | 0.182 | 0.195 | 0.324 | 0.183 | 0.197 | 0.322 | 0.13% | 0.14% | 0.21% | 0.67% |

**Table S4. Error evaluation of spatial transformation in Fig. 3b-c, (3).** To quantitively evaluate the 90^o^ rotation around the x-axis of the 5 selected points in Fig. 3b-c, (3), we present i) the calculated (cal.) values for the expected coordinates of x-axis 90^o^ rotation, ii) the output values for the remapped coordinates from the experimental results, and iii) the estimated PE and REE in the table. As estimated, PE is <0.68% and REE is <1.06% for the 5 selected points. PE and REE for the 867 coordinates of the 3D model are <1.48% and <2.39%, respectively, proving the high accuracy spatial transformation performed using our CNT tensor core.

| *Points* | *Cal. x-axis 90^o^ rotation* | | | *Output x-axis 90^o^ rotation* | | | *PE* | | | *REE* |
| --- | --- | --- | --- | --- | --- | --- | --- | --- | --- | --- |
|  | x | y | z | x’ | y’ | z’ | x-axis | y-axis | z-axis | – |
| 1 | 0.160 | 0.285 | 0.700 | 0.161 | 0.287 | 0.693 | 0.07% | 0.20% | 0.68% | 0.92% |
| 2 | 0.097 | 0.819 | 0.786 | 0.094 | 0.814 | 0.784 | 0.29% | 0.57% | 0.28% | 0.61% |
| 3 | 0.198 | 0.558 | 0.948 | 0.197 | 0.557 | 0.946 | 0.17% | 0.16% | 0.24% | 0.30% |
| 4 | 0.440 | 0.373 | 0.284 | 0.435 | 0.376 | 0.279 | 0.44% | 0.26% | 0.45% | 1.06% |
| 5 | 0.182 | 0.676 | 0.195 | 0.180 | 0.678 | 0.194 | 0.19% | 0.15% | 0.09% | 0.36% |

**Table S5**. Energy consumption for one scalar edge detection operation.

| *Category* | *Energy consumption per scalar operation (μJ)* | *Share of total energy consumption (%)* |
| --- | --- | --- |
| CNT NVMs | 0.0123 | 1.1 |
| TIA | 0.019 | 1.8 |
| Absolute | 0.484 | 44.6 |
| Summation | 0.569 | 52.5 |
| Total | 1.0843 | 100 |

**Movie S1.** Spatial transformation of 3D modal.

**Movie S2.** Edge detection of video flow.

**Supplementary References**

1	Schroder, D.K.. Series Resistance, Channel Length and Width, and Threshold Voltage. In *Semiconductor Material and Device Characterization*, D.K. Schroder (Ed.) (2005).

2	Pei, J. *et al.* Scalable Synaptic Transistor Memory from Solution-Processed Carbon Nanotubes for High-Speed Neuromorphic Data Processing. *Adv Mater* **37**, e2312783 (2025).

3	Gu, J. *et al.* Solution-Processable High-Purity Semiconducting SWCNTs for Large-Area Fabrication of High-Performance Thin-Film Transistors. *Small* **12**, 4993-4999 (2016).

4	Weng, J., Cohen, P. & Herniou, M. Camera calibration with distortion models and accuracy evaluation. *IEEE Transactions on Pattern Analysis and Machine Intelligence* **14**, 965-980 (1992).

5	Yogamani, S. *et al.* in *2019 IEEE/CVF International Conference on Computer Vision (ICCV)* 9307-9317 (2019).

6	Li, Y. *et al.* Memristors with analogue switching and high on/off ratios using a van der Waals metallic cathode. *Nature Electronics* **8**, 36-45 (2025).

7	Lu, X. F. *et al.* Exploring Low Power and Ultrafast Memristor on p-Type van der Waals SnS. *Nano Lett* **21**, 8800-8807 (2021).

8	Li, S. *et al.* Wafer-Scale 2D Hafnium Diselenide Based Memristor Crossbar Array for Energy-Efficient Neural Network Hardware. *Advanced Materials* **34**, 2103376 (2022).

9	Wu, X. *et al.* Novel nanocomposite-superlattices for low energy and high stability nanoscale phase-change memory. *Nature Communications* **15**, 13 (2024).

10	Lee, C. *et al.* Ultrahigh Stability and Operation Performance in Bi-doped GeTe/Sb2Te3 Superlattices Achieved by Tailoring Bonding and Structural Properties. *ACS Nano* **18**, 25625-25635 (2024).

11	Wang, S. *et al.* Two-dimensional ferroelectric channel transistors integrating ultra-fast memory and neural computing. *Nature Communications* **12**, 53 (2021).

12	Huo, J. *et al.* Coupled ferroelectric-anisotropic optoelectronic synapse for polarization-sensitive neuromorphic vision. *Nature Communications* **17**, 1468 (2026).

13	Zeng, J. *et al.* All-Ferroelectric Memtransistors for Brain-Inspired Computing. *Advanced Functional Materials* **36**, e31393 (2026).

14	Chen, J. *et al.* PZT-Enabled MoS2 Floating Gate Transistors: Overcoming Boltzmann Tyranny and Achieving Ultralow Energy Consumption for High-Accuracy Neuromorphic Computing. *Nano Letters* **23**, 10196-10204 (2023).
